# Identification of a locus associated with chlorosis and antioxidant capacity using RNA-seq and BSA-seq in soybean [*Glycine max* (L.) Merr]

**DOI:** 10.3389/fpls.2025.1598930

**Published:** 2026-06-29

**Authors:** Duo Lv, Lihua Zhu, Jiaqi You, Biting Cao, Hongjuan Yang, Weihong Gu, Qingzhu Li, Yuan Yuan, Chaohan Li

**Affiliations:** 1 Shanghai Key Laboratory of Protected Horticultural Technology, Horticultural Research Institute, Shanghai Academy of Agricultural Sciences, Shanghai, China; 2 Forestry and Pomology Research Institute, Protected Horticultural Research Institute, Shanghai Key Laboratory of Protected Horticultural Technology, Shanghai Academy of Agricultural Sciences, Shanghai, China

**Keywords:** *Glycine max*, etiolation, photosynthesis, bulked segregant analysis, 1-aminocyclopropane-1-carboxylate synthase

## Abstract

Plant coloration is an important trait in soybean (*Glycine max*), and its underlying molecular mechanisms are complex. In this study, we identified a chlorotic mutant, *el-5y*, in the wild-type soybean cultivar VS-5. Compared with those in VS-5, the capacities for photosynthesis, photosynthetic pigment synthesis, and antioxidant production in the *el-5y* mutant were significantly decreased, leading to a significant decrease in production. Transmission electron microscopy showed that the *el-5y* mutant cells were malformed, and abnormal lamellae and starch granules in the chloroplasts were significantly increased. Transcriptome analysis indicated that the differentially expressed genes (DEGs) between VS-5 and the *el-5y* mutants were mainly enriched in pathways related to the reduction–oxidation system, iron ion homeostasis, and auxin signaling transport. Based on the bulked segregant analysis data, the *el-5y* mutant was mapped to a 1.16-Mb interval on chromosome 11. The results of this study serve as a basis for further explorations into the molecular mechanisms that regulate plant coloration.

## Introduction

Soybeans (*Glycine max*) are the most important source of vegetative protein and oil globally, with an approximate average of 40% and 20% protein and oil contents, respectively (Zhang et al., 2015). Therefore, genetic improvements in soybean yield and seed quality are highly desirable. The yellow foliage phenotype of chlorotic soybean mutants is usually caused by defective cellular chlorophyll metabolism (Miao et al., 2016). Chlorophyll metabolism during photosynthesis is crucial for the productivity of green plants, as chlorophyll is essential for capturing light in the antenna systems and transferring energy within the reaction centers of photosynthesis (Liu et al., 2007). Therefore, mutants with chlorophyll variation, such as leaf-color mutants, serve as valuable models for fundamental research on photosynthesis (Wu et al., 2007; Li et al., 2019), photomorphogenesis (Parks and Quail, 1991), hormone physiology (Agrawal et al., 2001), resistance mechanisms, and the identification of gene function (Wu et al., 2007). Several genes related to the chlorotic phenotype of soybeans have been identified. GmCS1 (encoded by *Glyma20G179500*), a chorismate synthase, can affect photosynthesis by regulating chloroplast development. The leaves of the *Gmcs1* mutant were yellow–green variegated (Zhu et al., 2023). Mutations in two Mg-chelatase subunit genes, *GmChlI1a* (*Glyma13G30560*) and *GmChlI1b* (*Glyma15G08680*), caused a yellow foliage phenotype in soybean (Campbell et al., 2014). GmPsbP (*Glyma03G230300*) encoded an extrinsic protein of photosystem II, and a single base insertion in its second exon can result in a lethal yellow plant. Mutant genes related to etiolation considerably impact plant vitality (Sandhu et al., 2016). GmTic110 (*Glyma02G233700*) encodes a translocon in the inner chloroplast membrane that plays a critical role in plastid biogenesis (Sandhu et al., 2016).

Chloroplasts are not only sites for chlorophyll synthesis and photosynthesis but also crucial for auxin synthesis and reactive oxygen species (ROS) production (Yu et al., 2020). ROS are important signaling molecules in plant development and stress resistance. Generally, the production and clearance of ROS in cells are dynamic (Zhao et al., 2021; Chinnusamy et al., 2005). If the metabolism of ROS in plants is disturbed, ROS-mediated oxidative stress can cause various harmful cellular effects, such as plasmalemma peroxidation, nuclear damage, blocked photosynthesis, and abnormal respiration (Zaffagnini et al., 2019). In plant cells, there is a cross-interaction between ROS and multiple phytohormones. The development of lateral roots relies on the positive regulation between auxin transport signaling and ROS (Negi et al., 2008; Camacho-Cristóbal et al., 2015; Mangano et al., 2017); key genes influence the regulation of seed germination by ROS in the ABA signaling pathway (Zhang et al., 2019; Yang et al., 2018); salt stress increases endogenous ROS levels, leading to an elevation in 1-aminocyclopropane-1-carboxylic acid synthase (ACS) activity, thereby promoting the generation of ethylene (Zhu, 2000; Sarkar et al., 2018). Furthermore, the accumulation of ROS in cells can be regulated by the scavenging action of some terpenoid compounds, thereby protecting cells from oxidative damage and maintaining photosynthetic efficiency. Terpenoid compound production mainly depends on the plant’s mevalonate pathway (MVA).

In this study, we report a soybean chlorotic mutant (*el-5y*), a spontaneous mutant of the wild-type cultivar VS-5. The *el-5y* mutant showed yellowed cotyledons, leaves, and hypocotyls from the seedling stage. Although the leaves gradually turned green with development, the plant yellowed again when it entered the flowering stage. Simultaneously, the photosynthetic pigment content and various growth indices of the *el-5y* mutant were significantly lower than those of the VS-5. Based on bulked segregant analysis coupled with next-generation sequencing (BSA-seq), we localized the mutated gene to a region of approximately 1.16 Mb on chromosome 11, where only one gene had a non-synonymous mutation. Based on the transcriptome data, we hypothesized that the mutated gene promotes normal plant growth by maintaining the antioxidant defense capacity of the cell. This study contributes to our understanding of the molecular mechanisms underlying chloroplast development and leaf yellowing.

## Materials and methods

### Plant materials

*el-5y* is a spontaneous chlorotic mutant of VS-5 (wild type, a commonly cultivated variety). The F_1_ populations used to perform genetic analysis were derived from VS-5 and *el-5y*. Another commonly cultivated variety, Bayuebai, having the same field traits as VS-5, was used as the male parent and crossed with *el-5y* to produce an F_2_ population for mapping the *el-5y* locus in the spring of 2021 under natural photoperiodic conditions.

### RNA-seq and differentially expressed gene analysis

RNA for RNA-seq analysis was extracted from young leaves at the apical growth point during the early flowering stage. Comparative transcriptomic analysis was conducted using three biological replicates for each genotype at each developmental stage. The RNA samples were sequenced at Biomarker Bioinformatics Technology Co., Ltd. (Beijing, China), and for cDNA library construction, high-throughput sequencing (RNA-seq) was conducted using Illumina HiSeq™ 2500. During data verification, one forward-read (F1) file from a wild-type biological replicate at the first developmental stage was found to be corrupted and could not be recovered. Consequently, this replicate was excluded from the publicly deposited dataset, resulting in a total of 46 raw sequencing files. To evaluate the impact of this missing file, transcriptome analyses for the first developmental stage were repeated using the remaining two wild-type biological replicates together with three mutant biological replicates. The reanalysis reproduced the major findings and biological conclusions of the original study. The results of the reanalysis are provided in the Supplementary Materials (Table S2; Fig. S2 and S4). Raw sequencing data have been deposited in the Sequence Read Archive on the NCBI website, under the accession number PRJNA1455432.

The raw reads were first filtered to identify clean reads by removing those with only adaptor sequences and >5% unknown nucleotides and removing low-quality reads as well (percentage of low-quality bases with a quality value ≤5 in more than 50% of a read). The clean reads were mapped to the soybean genome sequence (Glycine_max.V1.0.dna.toplevel.fa) using the HISAT2 software (http://ccb.jhu.edu/software/hisat2/index.shtml). Based on the reference genome sequence, the mapped reads were joined using the StringTie software (Pertea et al., 2015).

Gene expression levels were analyzed using the method of fragments per kilobase of transcript per million (FPKM) mapped reads (Florea et al., 2013). Differential expression analysis between wild-type and mutant samples was performed using the edgeR software (Robinson et al., 2010). The resulting *p*-values were adjusted using the Benjamini–Hochberg approach to control the false discovery rate (FDR). Genes with an adjusted *p*-value < 0.05 and fold change ≥ 1.5 obtained by edgeR were selected as the thresholds for defining differentially expressed genes (DEGs).

### Transmission electron microscopy

Leaf segments (2 × 2 mm) of the wild type and *el-5y* mutants were fixed in 2.5% (w/v) glutaraldehyde, exposed to a vacuum for 10 min, and then rinsed with 0.1 M phosphate buffer. Subsequently, 1% Osmic acid was used to fix the samples and washed with 0.1 M phosphate buffer. The samples were then dehydrated using a gradient ethanol series [from 30% to 100% (v/v)] every 20 min. Finally, the samples were embedded in Spurr’s resin, cut using a LEICA UC6I microtome, and photographed using a JEM-123O scanning transmission electron microscope.

### Determination of photosynthetic pigment content and photosynthetic capacity

Samples were obtained from the cotyledons, true leaves at the rosette stage, and trifoliate leaves at different node positions using a hole puncher. The method described in a previous study (Tang et al., 2004) was used to assess the photosynthetic pigment content of leaves. Moreover, 80% acetone was used as the extraction solution, and absorbance values at 663.2, 646.8, and 470.0 nm were measured using a spectrophotometer to determine the photosynthetic pigment content. Each experiment comprised at least three biological replicates.

The net photosynthetic rate (*Pn*), stomatal conductance (*Gs*), intercellular carbon dioxide concentration (*Ci*), and transpiration rate (*Tr*) were measured from the seedling stage to the pod-bearing period on sunny mornings using a Li-6400 photosynthetic apparatus (Li-COR Company, Lincoln, NE, USA).

### Determination of physiological index

Superoxide dismutase (SOD), soluble protein, proline, and malondialdehyde (MDA) in trifoliate leaves from the second to seventh nodes were extracted using a standardized kit (Comin Biotechnology Co., Ltd., Suzhou, China), and their contents were measured using a spectrophotometer. Each experiment comprised at least three biological replicates.

### Bulked segregant analysis coupled with next-generation sequencing analysis

The genomic DNA of soybean was extracted from young leaves using the cetyltrimethylammonium bromide method (Miao et al., 2011). DNA quality was determined using a BioPhotometer Plus spectrophotometer (Eppendorf AG, Hamburg, Germany) and 1% agarose gel electrophoresis. The F_2_ population derived from Bayuebai crossed with *el-5y* was used for BSA-seq; four DNA pools were constructed by mixing equal amounts of DNA from 30 chlorotic individuals (yellow pool), 30 green individuals (green pool), Bayuebai, and *el-5y*. All DNA pools were sequenced on an illumina novaseq 6000 platform (illumina, USA). Raw data were deposited in the Sequence Read Archive on the NCBI website under accession number PRJNA1238100.

After sequencing, low-quality and short reads were removed, and clean reads were mapped to the reference soybean genome (Glycine_max.Wm82.a2.v1) using software (Li and Durbin, 2009). Then, the GATK software package (McKenna et al., 2010) was used to filter and purify single-nucleotide polymorphisms (SNPs) and insertions/deletions (InDels). To ensure the accuracy of mapping, the Euclidean distance (ED) algorithm (Lei et al., 2020; Hill et al., 2013) and the SNP index algorithm (Abe et al., 2012) were used to calculate the candidate regions of etiolation.

## Result

### *el-5y*, a spontaneous mutant, produces a chlorotic phenotype

**Figure 1:**
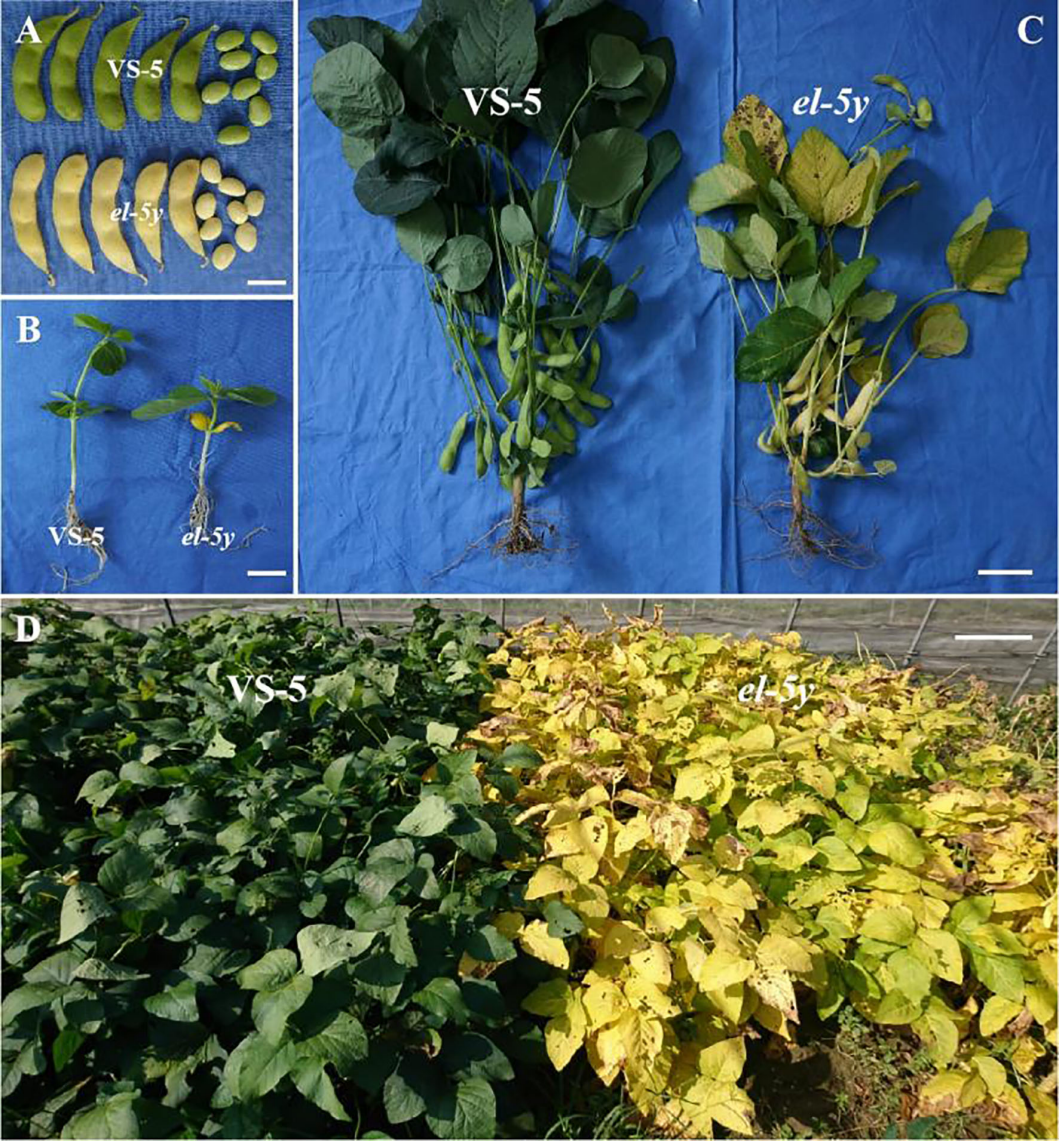
Comparison of main agronomic characters between VS-5 and *el-5y* mutant. **(A)** pods and seeds, scale bars is 2cm; **(B)** seeding stage, scale bars is 3cm; **(C)** Plants after R2 stage, scale bars is 4cm; **(D)** podding stage, scale bars is 10cm. Based on data from Li et al., 2019.

*el-5y* is a spontaneous VS-5 mutant. From VE to V2, cotyledons, hypocotyls, opposite leaves, and the first compound leaves of *el-5y* showed a “yellow” phenotype (**Figure 1B**). After entering the V3 stage, the leaves of the *el-5y* mutant gradually turned green (**Figure 1C**). However, when entering the R2 stage, the *el-5y* mutant rapidly exhibited yellowing throughout the plant and did not change to green thereafter; its fresh pods and fresh seed coats were yellow, whereas VS-5 remained green from VE to R6 (**Figures 1A, D**).

The differences between the mutant and wild-type plants not only are limited to plant color but also manifest in various agronomic traits during different stages of development (**Table 1**). Compared with those in VS-5, the average plant height, main stem diameter, and pod number per plant of the *el-5y* mutant decreased by approximately 30.13%, 4.46%, and 33.54%, respectively. Meanwhile, the pod weight per plant, 100-seed weight of fresh beans, and 100-seed weight of dried beans of the *el-5y* mutant also decreased by 45.64%, 13.48%, and 26.29%, respectively, compared with those in VS-5. However, we also found that the *vs-5y* mutant experienced more severe herbivory by pests in the field, indicating a significant decline in pest resistance (**Figures 1D**; **Supplementary Figure S1**).

**Table 1:** Comparison of main agronomic characters between VS-5 and *el-5y*. Based on data from Li et al., 2019.

Cultivars	Flower color	Pod color	Testa color	Plant height (cm)	Stem diameter (cm)	Node number	Branch number	Pod number per plant	Pod weight per plant (g)	100-seed weight of fresh bean (g)	100-seed weight of dried bean (g)
VS-5	White	Green	Light green	30.77 ± 4.51	1.12 ± 0.03*	7.33 ± 1.155	5.33 ± 0.58	42.63 ± 4.52	146 ± 12.46	103.20 ± 2.26*	42.83 ± 1.27*
*el-5y*	White	Light yellow	Yellow	21.50 ± 4.22	1.07 ± 0.02	7.00 ± 1.00	4.00 ± 1.00	28.33 ± 5.24	79.36 ± 7.21	89.72 ± 2.64	31.57 ± 3.11

*Significant differences between VS-5 and *el-5y* mutant (Student’s t-test, *p* < 0.05, n ≥ 4).

### *el-5y* mutant exhibits decreased pigment levels and photosynthetic performance

Plant coloration is closely related to pigment synthesis. To further clarify the reason for the “yellowed” phenotype, pigment content was measured in the *el-5y* mutant and VS-5 from stage V1 to R2 (**Table 2**; **Supplementary Table S1**). The results showed that the difference in chlorophyll content was positively correlated with the change in the color of *el-5y* plants. The contents of chlorophyll components in the cotyledons, opposite leaves, and the first two leaves of the *el-5y* mutant were significantly lower than those of VS-5 (**Table 2**). However, as the plants turned green, the difference in chlorophyll content between the *el-5y* mutant and VS-5 was no longer significant (**Supplementary Table S1**). Eventually, the *el-5y* mutant accumulated significant quantities of chlorophyll and turned green from stages V3 to R1. In R2, the chlorophyll content of *el-5y* decreased significantly (**Supplementary Table S1**). We also found that the chlorophyll *a*/*b* ratio in most leaves of *el-5y* was greater than that in VS-5, indicating that chlorophyll *b* synthesis was more severely affected in the *el-5y* mutant (**Table 2**; **Supplementary Table S1**). By contrast, the carotenoid content in the *el-5y* mutant was always lower than that in VS-5 at different developmental stages.

**Table 2:** Comparison of chloroplast pigment contents in different parts of tissues between VS-5 and *el-5y* mutant. Based on data from Li et al., 2019.

Cultivars	Tissues	Chlorophyll *a* (μg/g FW)	Chlorophyll *a* (μg/g FW)	Carotenoid *a* (μg/g FW)	Total pigments (μg/g FW)	Chlorophyll *a*/chlorophyll *b*
VS-5	Cotyledon	250.26 ± 18.37*	110.47 ± 10.38*	54.82 ± 0.86*	415.54 ± 28.64*	2.27 ± 0.05*
	Opposite leaf	1,862.19 ± 101.41*	637.97 ± 38.08*	425.28 ± 20.34*	2,925.44 ± 159.70*	2.92 ± 0.02
	Tri-foliolate	1,738.25 ± 139.87*	594.12 ± 44.59*	449.31 ± 36.55*	2,781.68 ± 219.76*	2.93 ± 0.05*
***el-5y***	Cotyledon	14.05 ± 5.27	16.11 ± 4.8	13.46 ± 0.15	43.62 ± 10.21	0.86 ± 0.06
	Opposite leaf	1,398.46 ± 106.87	435.09 ± 27.22	364.43 ± 30.79	2,197.98 ± 164.83	3.21 ± 0.05*
	Tri-foliolate	1,055.24 ± 103.32	368.60 ± 43.29	319.48 ± 25.09	1,743.31 ± 171.67	2.87 ± 0.05

*Significant differences between VS-5 and *el-5y* mutant (Student’s t-test, *p* < 0.05, n ≥ 4).

Photosynthesis is the primary driving force behind the growth of aboveground plant parts. To investigate the impact of reduced chlorophyll on the development and growth of plants in the *el-5y* mutant, the photosynthetic capacity and physiological characteristics of both VS-5 and the *el-5y* mutants were assessed (**Table 3**). Compared to those in VS-5, the net photosynthetic rate (*Pn*) and transpiration rate (*Tr*) of the second section of leaves of the *el-5y* mutant significantly decreased by 12.07% and 33.87%, respectively, whereas the intercellular concentration of CO_2_ (*Ci*) increased by 24.59%. As the *el-5y* plant gradually turned green, the differences in the *Pn*, *Gs*, and *Ci* of the third section of leaves between VS-5 and the *el-5y* mutants were not significant, and only the *Tr* of *el-5y* was lower than that of VS-5. However, when the *el-5y* mutant plants underwent yellowing again, there was a sharp decline in *Pn*, *Gs*, and *Tr*, whereas *Ci* increased significantly. Compared with those in VS-5, the *Pn* of the sixth and seventh leaf sections of the *el-5y* mutant decreased by 41.59% and 69.64%, respectively. Similarly, the *Gs* of the sixth and seventh leaf sections decreased by 51.62% and 70.21%, and *Tr* decreased by 38.59% and 35.24% in the *el-5y* mutant, respectively. Meanwhile, the *Ci* of the sixth and seventh leaf sections of the *el-5y* mutant increased by 100.67% and 72.60%, respectively, when compared to that in VS-5, respectively.

**Table 3:** Comparison of chloroplast pigment contents in different parts of tissues between VS-5 and *el-5y* mutant. Based on data from Li et al., 2019.

Cultivars	Node order	*Pn* (μmol·m^−2^·S^−1^)	*eGs* (μmol·m^−2^·S^−1^)	*Ci* (μL·L^−1^)	*Tr* (mmol·m^−2^·S^−1^)
VS-5	2	16.07 ± 0.65*	356.00 ± 13.53	329.33 ± 13.01	6.26 ± 0.16*
	3	17.43 ± 0.87	422.33 ± 34.08	243.33 ± 10.79	8.90 ± 0.21*
	4	20.83 ± 1.11*	517.33 ± 29.96	224.33 ± 27.02	9.49 ± 0.37*
	5	23.83 ± 1.27*	740.33 ± 28.71*	214.67 ± 27.57	10.90 ± 0.37*
	6	19.40 ± 0.80*	707.67 ± 20.03*	199.33 ± 17.10	9.25 ± 0.37*
	7	16.57 ± 0.87*	624.33 ± 15.63*	264.00 ± 28.05	8.23 ± 0.10*
*el-5y*	2	14.13 ± 0.40	316.00 ± 16.37	410.33 ± 12.01	4.14 ± 0.10
	3	15.80 ± 0.40	365.33 ± 22.55	323.67 ± 14.98*	6.93 ± 0.27
	4	18.23 ± 0.75	444.67 ± 20.79	269.67 ± 26.31	8.16 ± 0.28
	5	19.13 ± 0.74	615.00 ± 31.76	228.67 ± 26.69	8.96 ± 0.37
	6	11.33 ± 1.01	342.33 ± 27.10	402.00 ± 19.97*	5.68 ± 0.06
	7	5.03 ± 0.31	186.00 ± 7.21	455.67 ± 10.97*	5.33 ± 0.26

*Pn*, net photosynthetic rate; *Gs*, stomatal conductance; *Ci*, intercellular CO_2_ concentration; *Tr*, transpiration rate.

*Significant differences between VS-5 and *el-5y* mutant (Student’s t-test, *p* < 0.05, n ≥ 3).

### RNA-seq of soybeans from different development stages

To better explore the molecular mechanisms underlying the phenotypic differences between VS-5 and the *el-5y* mutants, RNA-seq was performed on soybean leaves at different developmental stages. Approximately 5.71-GB bases were generated from each sample using the Illumina HiSeq platform. After mapping the sequenced reads to the reference genome (soybean Wm82.a2 genome, https://www.soybase.org), 59,760 genes were obtained from all samples, including 3,716 newly identified genes. The quality of the RNA-seq data is summarized in **Table 4**. The correlations between the samples from different stages and materials were also calculated to test whether the chosen samples were reliable. Biological replicates at different stages showed a good correlation (**Supplementary Figure S2**).

**Table 4:** Summary of RNA-seq data.

Samples		Clean reads	Clean bases	GC content	≥Q30 (%)	Total reads (%)	Mapped reads (%)	Uniq mapped reads (%)	Multiple map reads (%)
VY-5-1	a	20,798,217	6,224,023,024	46.33	92.90	41,596,434	95.12	90.30	4.82
	b	20,811,984	6,220,594,242	46.41	93.09	41,623,968	94.90	90.36	4.54
	c	23,440,287	7,008,870,044	46.39	93.21	46,880,574	94.78	90.11	4.67
VY-5-2	a	22,770,813	6,806,368,760	46.10	92.08	45,541,626	94.69	91.71	2.98
	b	21,951,971	6,561,917,972	46.13	92.00	43,903,942	94.14	91.10	3.04
	c	21,102,628	6,308,488,648	45.73	92.30	42,205,256	94.47	91.48	2.99
VY-5-3	a	20,484,940	6,133,223,586	46.79	92.81	40,969,880	95.32	90.62	4.71
	b	19,711,225	5,894,136,212	46.82	93.04	39,422,450	95.07	90.40	4.67
	c	21,503,918	6,429,737,724	46.67	93.09	43,007,836	95.03	90.47	4.55
VY-5-4	a	20,695,669	6,189,265,436	46.01	92.88	41,391,338	94.60	87.39	7.21
	b	21,582,593	6,450,788,646	45.94	92.81	43,165,186	94.55	88.95	5.60
	c	22,801,496	6,826,831,306	46.17	92.75	45,602,992	95.04	86.41	8.64
*el-5y*-1	a	21,008,321	6,282,099,332	46.32	93.09	42,016,642	94.84	89.82	5.02
	b	20,256,731	5,992,493,916	45.75	93.05	40,513,462	93.79	89.03	4.76
	c	24,062,569	7,196,314,892	45.91	92.90	48,125,138	93.24	88.47	4.77
*el-5y*-2	a	21,296,691	6,366,165,856	45.87	92.66	42,593,382	94.94	91.81	3.13
	b	21,698,653	6,489,428,128	46.33	92.29	43,397,306	95.17	91.91	3.25
	c	21,394,099	6,393,580,968	46.23	93.04	42,788,198	95.12	91.82	3.30
*el-5y*-3	a	23,373,274	6,987,940,066	46.60	92.83	46,746,548	94.66	90.27	4.39
	b	23,071,584	6,893,700,016	46.27	92.13	46,143,168	94.48	90.30	4.18
	c	27,210,997	8,126,971,204	46.37	93.13	54,421,994	94.66	90.29	4.37
*el-5y*-4	a	19,783,845	5,920,041,190	45.62	92.76	39,567,690	94.11	87.49	6.63
	b	20,221,671	6,040,475,576	46.01	92.48	40,443,342	93.54	87.12	6.42
	c	19,075,936	5,706,744,002	45.24	92.75	38,151,872	94.48	91.38	3.10

GC content refers to the proportion of guanine (G) and cytosine (C) nucleotides in a nucleic acid sequence.

Using the thresholds of an FDR < 0.05 and fold change of expression level ≥1.5, the transcriptome data were analyzed by comparing FPKM values from different libraries (**Supplementary Table S2**). For the leaves at the second node (the *el-5y* mutant is yellow), 1,276 DEGs were identified between VS-5 and the *el-5y* mutants, comprising 613 upregulated and 663 downregulated genes in the *el-5y* mutant. For leaves in the fourth node (the *el-5y* mutant is green), only 427 DEGs were identified between VS-5 and the *el-5y* mutants, comprising 205 upregulated and 222 downregulated genes in the *el-5y* mutant. For the leaves at the sixth node (the *el-5y* mutant was yellowed again), 940 DEGs were identified between VS-5 and the *el-5y* mutants, of which 376 were downregulated and 564 were upregulated in the *el-5y* mutant. In fresh seeds (the *el-5y* mutant was yellowed), 807 DEGs were identified between VS-5 and the *el-5y* mutants, comprising 534 upregulated and 273 downregulated genes in the *el-5y* mutant.

### Comparative transcriptome analysis between the wild type and *el-5y* mutants

**Figure 2:**
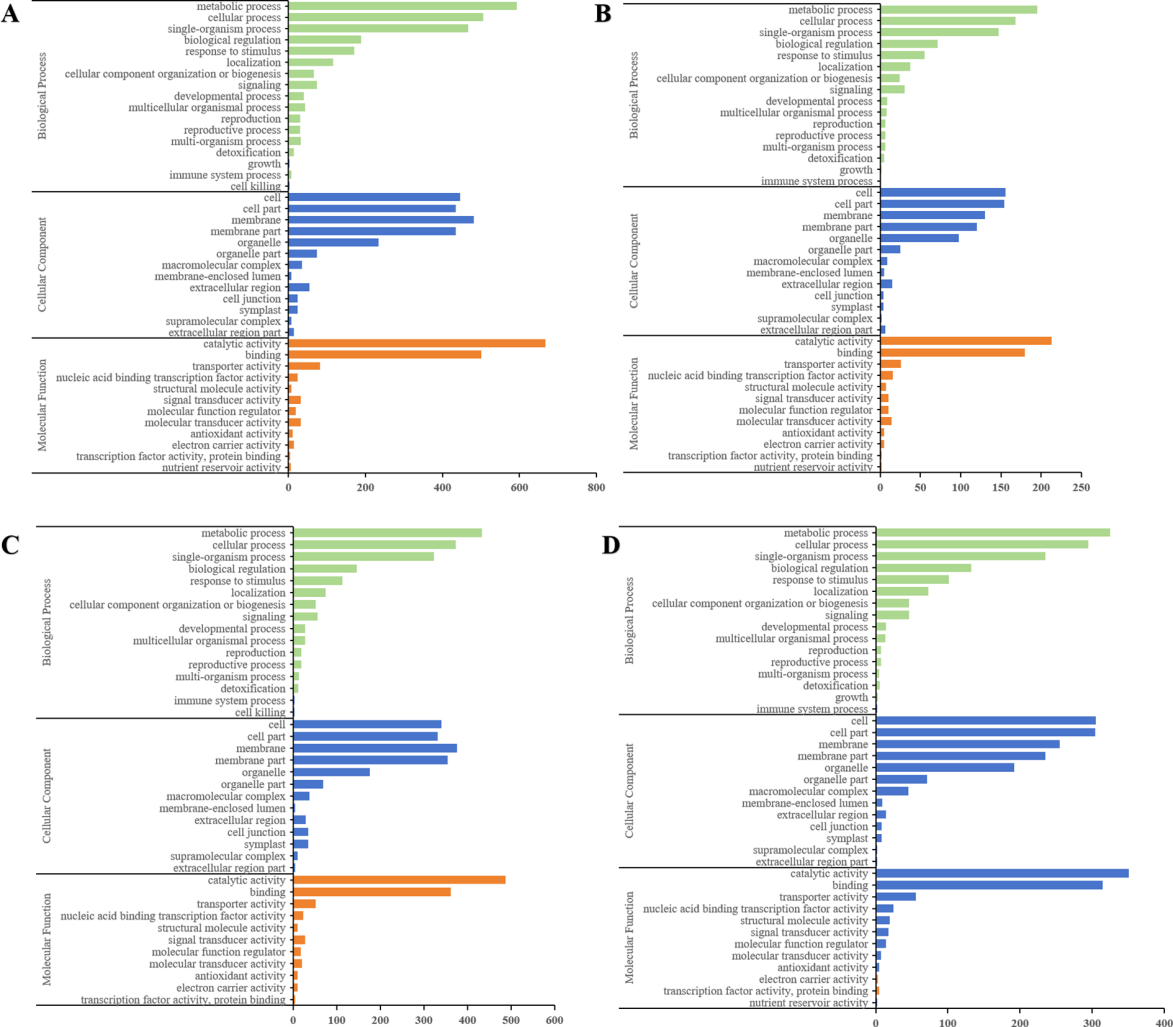
Histogram presentation of Gene Ontology (GO) classifications. x-axis indicates the number of DEGs in each subcategory. y-axis indicates the GO subcategories. **(A)** DEGs from the leaves at the second node; **(B)** DEGs from the leaves at the fourth node; **(C)** DEGs from the leaves at the sixth node; **(D)** DEGs from fresh seeds.

**Figure 3:**
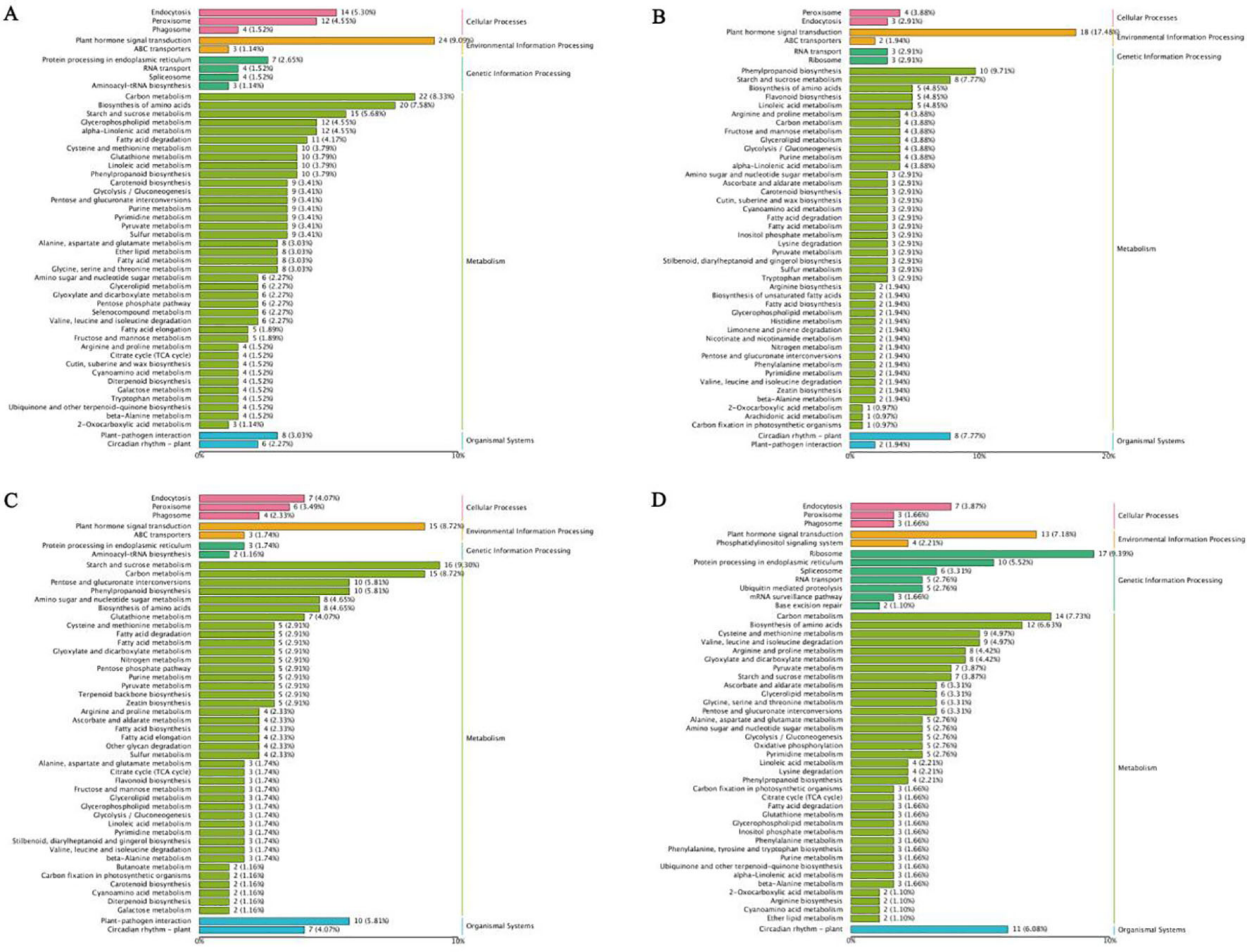
Histogram presentation of Kyoto Encyclopedia of Genes and Genomes (KEGG) classifications. y-axis indicates the KEGG subcategories; the numbers in parentheses indicates the number of DEGs in each subcategory; x-axis and the numbers outside parentheses indicates percentage of DEGs in the subcategories. **(A)** DEGs from the leaves at the second node; **(B)** DEGs from the leaves at the fourth node; **(C)** DEGs from the leaves at the sixth node; **(D)** DEGs from fresh seeds.

To evaluate the functional categories of DEGs between VS-5 and the *el-5y* mutants at different developmental stages, we performed Gene Ontology (GO) and Kyoto Encyclopedia of Genes and Genomes (KEGG) analyses (**Figures 2**, **3**, **Supplementary Figures S3, S4**).

Overall, the annotation results from both databases showed that the DEGs in leaves at different developmental phases were significantly enriched in pathways related to plant hormone signal transduction, endocytosis, carbohydrate transmembrane transport, and REDOX processes. In the plant hormone signal transduction category, DEGs related to auxin transport and metabolism were the most abundant (**Supplementary Table S3**). Furthermore, compared with those in VS-5, DEGs that were upregulated or downregulated during the two yellowing phases in the *el-5y* mutant were also enriched in pathways related to iron ion homeostasis. These results indicated that the mutation in the *el-5y* mutant had a sustained impact on cellular REDOX processes and the auxin pathway. However, compared to that in the two yellowing phases, when leaf greening occurred in the *el-5y* mutant, although GO analysis revealed that some DEGs were enriched in pathways related to the cellular REDOX process, their numbers were significantly reduced. Correlation analysis showed that the transcriptome data from fresh seeds in both VS-5 and the *el-5y* mutants had a low correlation with the transcriptome data from leaves (**Supplementary Figure S2**). However, DEGs in the fresh seeds of VS-5 and the *el-5y* mutants were also significantly enriched in pathways related to plant hormone signal transduction, and most were auxin-responsive proteins (**Figures 2D**, **3D**). Compared with those in the previous two yellow phases, DEGs in fresh seeds were no longer enriched in pathways related to the REDOX process but were enriched in protein processing and amino acid metabolism. Furthermore, the GO analysis showed that DEGs were enriched in secondary metabolite biosynthetic processes. Seed development is accompanied by the production and accumulation of numerous nutrients and substances. The above results showed that the mutation in the *el-5y* mutant affects the cellular REDOX process and seed development in plants.

Next, we focused on 75 DEGs that exhibited consistent expression patterns throughout all three leaf developmental phases (**Supplementary Table S2**). These DEGs were likely directly regulated by mutations in the *el-5y* mutant. Even when the expression levels of many other DEGs return to normal, their constant differential expression may lead to chlorosis in the *el-5y* mutant. GO analyses indicated that these 75 DEGs were primarily enriched in auxin catabolic processes, small GTPase-mediated signal transduction, peptidyl-tyrosine phosphorylation, iron ion transport, and homeostasis (**Supplementary Figure S5**).

To validate the accuracy of the RNA-seq data, qRT-PCR was conducted on six randomly selected DEGs from the RNA-seq data to detect their expression patterns at the three developmental stages. The results showed a high expression pattern correlation between the RNA-seq data and qRT-PCR in all selected genes, whether in VS-5 or mutants (R = 0.91–0.99; **Supplementary Figure S6**).

### The *el-5y* mutant has defective antioxidant capacity and cell morphology

Transcriptome data showed that the DEGs were mainly enriched in pathways related to cellular REDOX processes. Considering that the *el-5y* mutant plants exhibited poor growth, this finding suggested that the antioxidant system of the *el-5y* mutant cells may be unbalanced. To test this hypothesis, we measured the physiological characteristics of both the wild-type and mutant plants.

**Figure 4:**
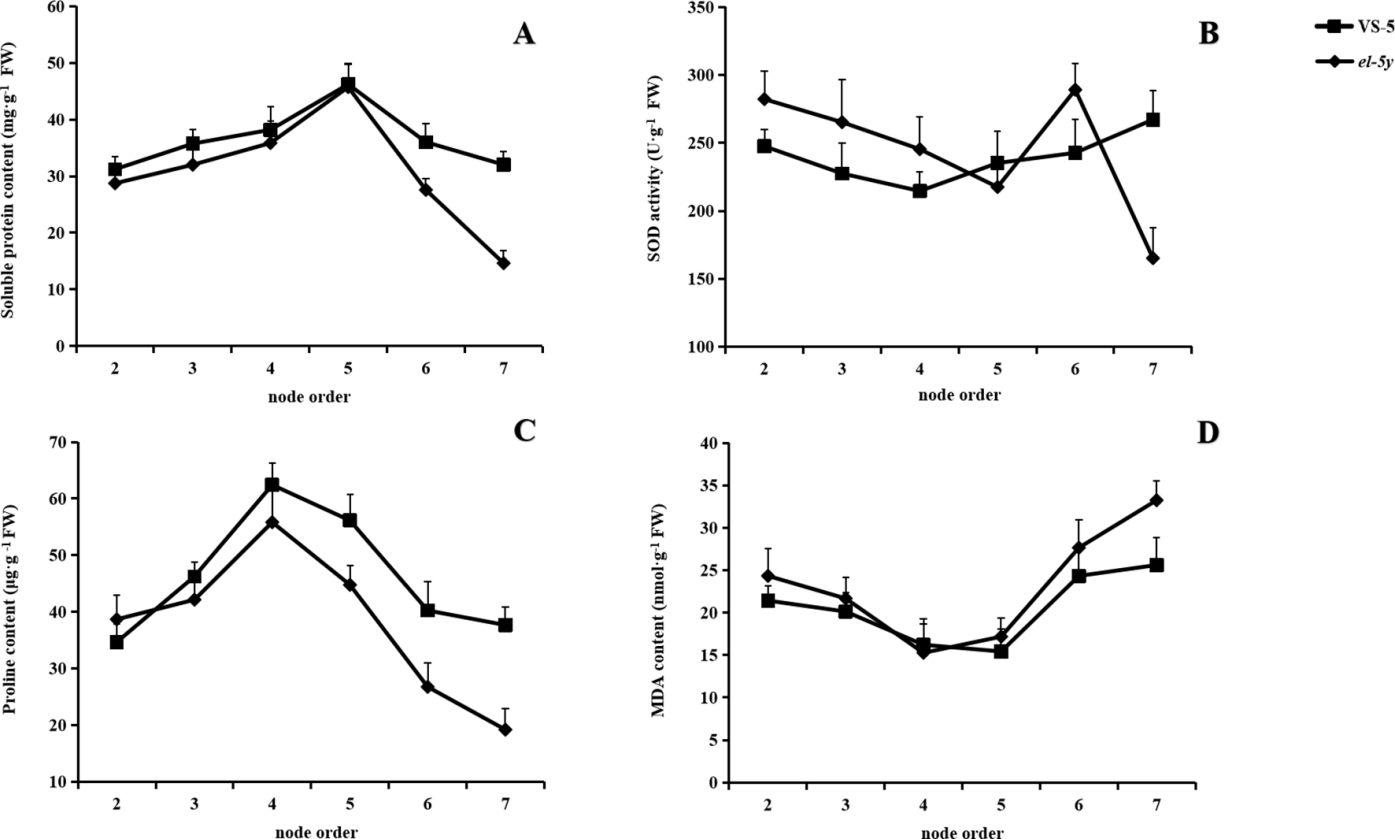
**(A-D)** Comparison of multiple physiological indicators between VS-5 and el-*5y* mutant. Based on data from Li et al., 2019.

The soluble protein content (SPC) in the leaves of VS-5 and the *el-5y* mutants gradually increased as the plants grew, but gradually decreased as the plants entered the reproductive growth stage (leaves at the sixth and seventh nodes; **Figure 4A**). However, compared to VS-5, the *el-5y* mutant showed a lower SPC at every growth stage, especially at the sixth and seventh nodes, where the SPC of *el-5y* decreased sharply. Similar to the SPC, the proline content in the leaves of both VS-5 and the *el-5y* mutants increased as the plants grew; however, when the plants entered the flowering stage, the proline content began to decrease significantly (**Figure 4C**). The SOD activity in the leaves of the *el-5y* mutant was significantly higher than that of VS-5 before podding, but sharply decreased in the *el-5y* mutant once podding began (**Figure 4B**). Unlike other physiological indices, the MDA content of the *el-5y* mutant remained similar to that of VS-5 throughout the growth process, and even the MDA content of *el-5y* was significantly higher than that of VS-5 when plants entered the reproductive growth stage (**Figure 4D**).

**Figure 5:**
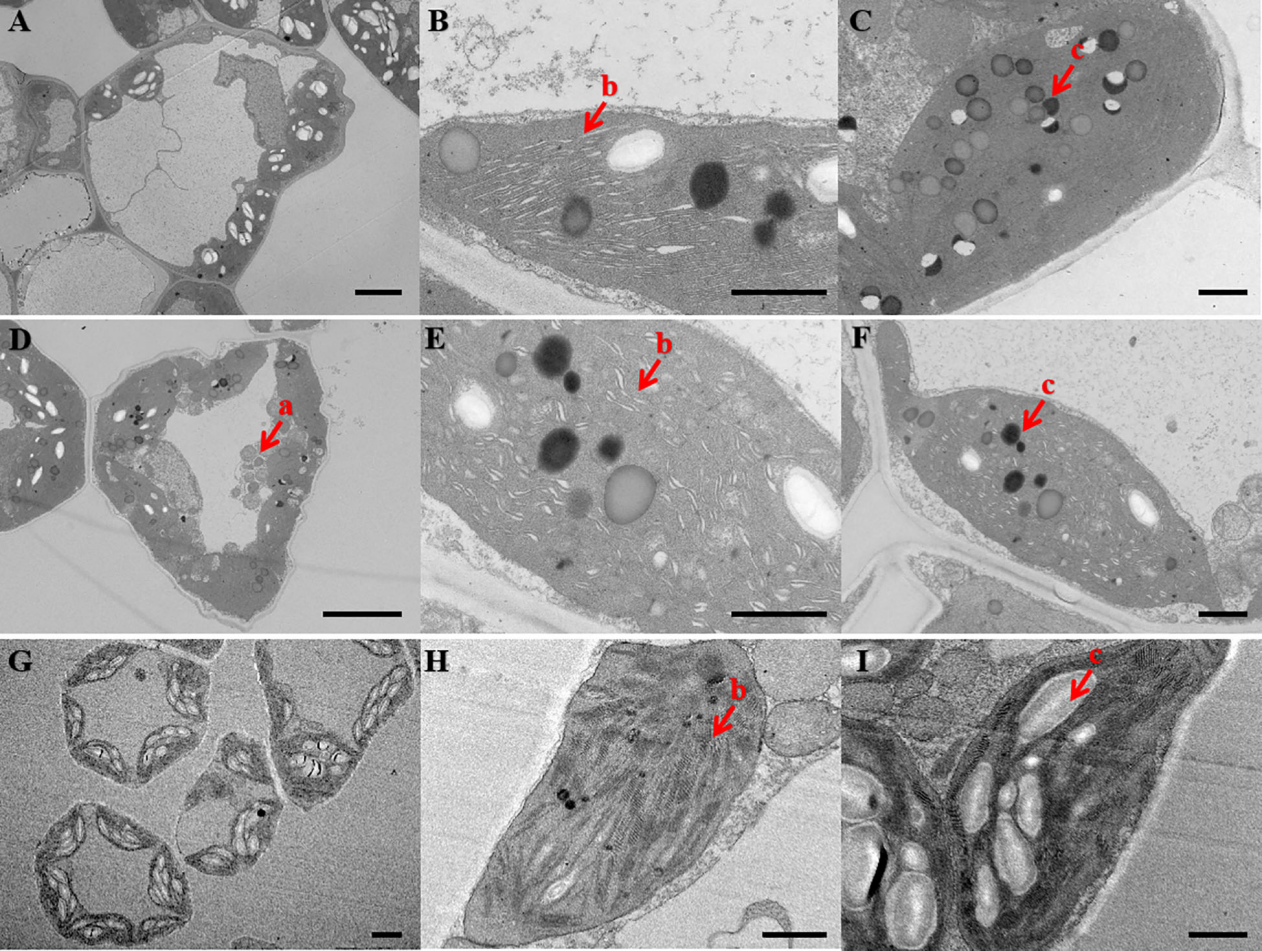
Transmission electron microscopy (TEM) of leaf cells in VS-5 and *el-5y* mutant. **(A–C)** Light microscopy image; **(D–F)** yellowed leaf cells in *el-5y* mutant; **(G–I)** green leaf cells in *el-5y* mutant; **(A, D, G)** entire cell, scale bars are 50μm; **(B, C, E, F, H, I)** chloroplast, scale bars are 10μm; **a** insoluble plastids in cytoplasm, **b** Thylakoids, **c** starch grains.

The imbalance in the antioxidant system also led to oxidative damage in the *el-5y* mutant cells. Transmission electron microscopy (TEM) showed that during the yellowed stages, the leaf cells of the *el-5y* mutant shrunk, the membrane system was invaginated, and some insoluble material appeared inside the cells (**Figures 5D–F**). Additionally, the chloroplasts exhibited severe deformation in the leaf cells of the *el-5y* mutant, with twisted thylakoid layers and a significant reduction in starch grain number. However, when the plants turned green, the leaf cells of the *el-5y* mutant resembled those of the wild type, with no further shrinkage or well-developed chloroplast structures (**Figures 5G–I**).

### Multiple phytohormone content analysis between wild type and *el-5y* mutants

Transcriptome data showed that VS-5 and the *el-5y* mutants exhibited significant differences in several phytohormone signaling pathways. To further investigate the impact of these DEGs on the different phytohormone pathways, we selected hypocotyls (green in VS-5 and yellowed in the *el-5y* mutant) and green leaves (green in both VS-5 and the *el-5y* mutant) for phytohormone content analysis (**Table 5**). The results showed that the content of free auxin (IAA) in the yellowing hypocotyls of the *el-5y* mutant (5.54 ng/g) was significantly lower than that of VS-5 (9.93 ng/g), while the content of conjugated auxin (IAA-ASP and IAA-GLU) was slightly higher in the *el-5y* mutant (20.61 ng/g) than in VS-5 (18.28 ng/g). However, there was no significant difference in the content of auxin synthesis precursors [indole-3-acetamide (IAM) and thioacetamide (TAM)] between VS-5 and the *el-5y* mutants. When the plants turned green, we found that the free auxin content in the *el-5y* mutant leaves (170.83 ng/g) was significantly higher than in VS-5 (132.34 ng/g), while the content of conjugated auxin was lower in the mutant than in VS-5. Additionally, the auxin precursor content in the *el-5y* mutant leaves (50.37 ng/g) was significantly lower than that in VS-5 (60.78 ng/g). These results suggested that leaf color is closely related to the auxin pathway, and when the *el-5y* mutant turned green, the efficiency of auxin utilization increased significantly.

**Table 5:** Comparison of various phytohormone contents between VS-5 and *el-5y* mutant.

Tissues	Samples	IAA	IAA-ASP	IAA-GLU	IAM	TAM	SA	SAG	JA	MEJA	JA-ILE	OPDA	ABA
Green leaf	VS-5	132.34	221.2*	66.0*	1.7	58.7*	614.4	61.9	327.9*	2.4	19.4	190.7	230.5*
	el-5y	170.83*	199.2	53.8	2.0	46.5	772.8*	197.0*	292.2	2.3	26.9*	321.7*	162.10
Yellowed hypocotyl	VS-5	9.93*	17.3	1.00	0.6	7.5	532.6*	1,930.8	30.1*	NA	0.6	132.93*	59.0*
	el-5y	5.54	19.5*	1.16	0.2	7.8	282.8	4,202.0*	23.02	NA	0.8	51.5	11.3

IAA, indole-3-acetic acid; IAA-ASP, indole-3-acetic acid aspartate; IAA-GLU, indole-3-acetic acid glutamate; IAM, indole-3-acetamide; TAM, thioacetamide; SA, salicylic acid; SAG, salicylic acid glucoside; JA, jasmonic acid; MEJA, methyl jasmonate; JA-ILE, jasmonyl-isoleucine; OPDA, 12-oxo-phytodienoic acid; ABA, abscisic acid.

*Significant differences between VS-5 and el-5y mutant (Student’s t-test, p < 0.05, n ≥ 4).

By contrast, the conjugated salicylic acid glucoside (SAG) content in the *el-5y* mutant was consistently higher than that in VS-5 in different tissues. In the yellowed hypocotyls of the *el-5y* mutant (282.80 ng/g), the free salicylic acid (SA) content was significantly lower than that of VS-5 (532.62 ng/g). However, after the plants turned green, the free SA content in the *el-5y* mutant leaves (772.87 ng/g) was significantly higher than that in VS-5 (614.37 ng/g). This finding suggested that when the leaves turned green, the ability to utilize conjugated SA was enhanced in the *el-5y* mutant.

In addition, the free JA content in VS-5 was consistently higher than that in the *el-5y* mutant. Similarly, the JA synthesis precursor [12-oxo-phytodienoic acid (OPDA)] content in the *el-5y* mutant hypocotyls (51.54 ng/g) was lower than that in VS-5 (132.93 ng/g). However, in the green leaves, the OPDA content of the *el-5y* mutant (321.67 ng/g) was higher than that in VS-5 (190.69 ng/g). These results indicated that in yellowed tissues, the JA synthesis pathway in the *el-5y* mutant was inhibited. However, after the leaves turned green, the JA synthesis pathway in the *el-5y* mutant was recovered, although the utilization pathway of its precursor was still blocked. The ABA content in the *el-5y* mutant was consistently higher than that in VS-5, and this trend remained unchanged, regardless of the color change in the plant.

### Genetic analysis and preliminary mapping of the mutation in the *el-5y* mutant

*el-5y* is a spontaneous chlorotic mutant derived from VS-5, and significant phenotypic differences between VS-5 and the *el-5y* mutants were likely caused by a mutation in a single gene. To analyze the genetic inheritance pattern of the mutant locus and achieve further mapping, reciprocal crosses were performed between the *el-5y* mutant and VS-5 to produce an F_1_ population. Additionally, another cultivated variety, Bayuebai, with the same phenotype as VS-5, was used as the male parent and crossed with an *el-5y* mutant to produce an F_2_ population.

In the F_1_ individuals, the phenotype was consistent with that of VS-5, and no significant morphological differences were found between the homozygous dominant individuals. In the F_2_ population with 140 individuals generated from the *el-5y* mutant and Bayuebai, 119 and 31 plants exhibited the Bayuebai and *el-5y* phenotypes, respectively. It fitted a 3:1 segregation ratio (χ^2^ = 2.33 < χ^20.05^ = 3.84, *p* = 0.13; **Table 6**). These results indicated that the *el-5y* locus is conferred by a single recessive nuclear gene in soybeans.

**Table 6:** Genetic analysis of the mutant locus in *el-5y* mutant.

Population	Total number	Green leaf	Yellowed leaf	Expected ratio	χ^2^
F_1_	20	20	0	/	/
F_2_	140	119	31	3:1	2.33

χ^2^ (0.05,1) = 3.84.

The BSA method was used for the genetic mapping of the mutant locus. In total, 31.57, 41.22, 113.25, and 120.38 Gb of raw data were generated for the *el-5y*, Bayuebai, green pool, and yellow pool, representing approximately 19×, 18×, 33×, and 29× genome coverage, respectively. The filtered clean reads of each sample were mapped to the reference genome of the soybean cultivar, and 275,831 and 59,281 non-synonymous SNPs/InDels were identified in the *el-5y* mutant vs. Bayuebai and green pool vs. yellow pool, respectively.

To obtain the candidate region associated with the chlorotic phenotype, two approaches, the ΔSNP-index and ED algorithms, were used to calculate the allele segregation of the SNPs and InDels between the two extreme DNA pools. Both methods showed that the region most closely associated with the chlorotic phenotype was on chromosome 11 (Chr.11) and overlapped significantly. Finally, the 1.16-Mb region (0.98–2.14 Mb) on Chr.11 was identified as the candidate region for the chlorotic phenotype (**Supplementary Figure S7**). In the 1.16-Mb interval, there were 227 genes, but only six had non-synonymous mutations in the *el-5y* mutant. Owing to the lack of a sufficiently large genetic population, the candidate gene could not be fine-mapped temporarily; however, by analyzing the function and effect of the mutation site of the six genes (**Table 7**), we speculated that *Glyma11G028200* was the candidate gene for the mutation. This was because, among the six genes, only the mutant sites of *Glyma11G028200* occurred in the functional domain of the protein (**Supplementary Figure S8**). Additionally, the product encoded by *Glyma11G028200* is 1-aminocyclopropane-1-carboxylate synthase (ACS). Several studies have shown that endogenous ACS activity in plants is closely related to cellular ROS levels and auxin transport (Liu et al., 1985; Sandalio et al., 2016). In chlorotic soybean seedlings of *Phaseolus radiatus* L., exogenous IAA can cause an increase in endogenous ROS levels, thereby enhancing ASC activity in the cells (Ke and Sun, 2004).

**Table 7:** Genes with nonsynonymous mutations in candidate region.

Gene ID	Homologs	Mutant form		Mutation site on the protein sequence	Function	Major domain is mutated
*Glyma11G027800*	*AT4G11970*	aCt/aTt T/I		18	YTH family protein	No
*Glyma11G028200*	*AT1G62960*	aCt/aGt T/S	aCc/aTc T/I	137, 251	ACC synthase	Yes
*Glyma11G028500*	*AT4G22840*	Aac/Cac N/H	Aac/Cac N/H	12, 40	Sodium bile acid symporter	No
*Glyma11G028600*	*AT4G22830*	Caa/Aaa Q/K		39	YCF49-like protein	No
*Glyma11G028900*	*AT4G22790*	cTc/cCc L/P	Cag/Aag Q/K	400, 437	MATE efflux family protein	No
*Glyma11G029400*	*AT1G62870*	gAc/gGc E/D		460	Uncharacterized protein	No

## Discussion

As chlorotic mutants are usually accompanied by a loss of plant vitality (Miao et al., 2016; Sandhu et al., 2016), studies that have focused on the mapping and cloning of genes related to the yellowing phenotype have always attracted considerable research attention. Until now, there have been many studies on chlorotic mutants in plants, wheat (*Triticum aestivum* L.) (Hui et al., 2012), *Arabidopsis thaliana* (Nagata et al., 2005), rice (Nagata et al., 2005; Zhang et al., 2013), maize (Guan et al., 2016), carrot (*Daucus carota* L.) (Budahn et al., 2014), cucumber (Miao et al., 2016; Xiong et al., 2020), and many other crops (Barry et al., 2008; Zhu et al., 2020). In the present study, based on the analysis of BSA-seq and transcriptome data, we mapped a locus that can cause a chlorotic phenotype in soybean and speculated that *Glyma11G028200*, which encodes ACS, is the candidate gene.

Chlorotic mutants are typically associated with chloroplast development and chlorophyll synthesis (Xiong et al., 2020), which can result in weaker photosynthetic capacity. To determine whether the yellowing phenotype of the *el-5y* mutant was also related to chloroplast development, we measured the photosynthetic pigment content and photosynthetic capacity parameters in both the *el-5y* mutant and the wild type (VS-5). Compared to those in VS-5, the total content of photosynthetic pigments, the content of different types of photosynthetic pigments, and the net photosynthetic rate of the *el-5y* mutant were significantly lower. Ultrastructural analysis using TEM showed that the cell structure of the *el-5y* mutant was deformed, resembling a state of aging and dehydration. The biomembrane of the *el-5y* mutant cell was severely invaginated and contained insoluble plastids in the cytoplasm. In particular, in the chloroplasts of the *el-5y* mutant, the number of abnormal lamellae and starch grains increased significantly, which disturbed the synthesis of chlorophyll and the photosynthetic capacity in the *el-5y* mutant cells. These results suggested that the function of mutated genes in the *el-5y* mutant should not be limited to affecting chloroplast development but should also impact the development of the entire cell.

The plant antioxidant system (including soluble proteins, proline, and a series of antioxidant enzymes) plays an important role in clearing ROS during stress resistance and anti-aging processes (Zhao et al., 2021; Zhu, 2000). If the balance between ROS production and clearance is disrupted, excessive ROS accumulation can increase lipid peroxidation, resulting in MDA accumulation and chlorophyll degradation (Zhu, 2000). Compared to those in VS-5, the *el-5y* mutant exhibited higher levels of SOD content in the early stage of development (especially when the *el-5y* mutant showed a “yellowing” phenotype), but as the plants begin to pod, the SOD content in the *el-5y* mutant decreased rapidly. Additionally, the soluble protein and proline contents in the *el-5y* mutant were slightly lower than those in VS-5 during the early stage of development; however, this difference between VS-5 and the *el-5y* mutants became increasingly significant as the plants began to pod. Furthermore, the MDA content of the *el-5y* mutant was significantly higher than that of VS-5 when the *el-5y* mutant showed a “yellowing” phenotype. These results are consistent with the transcriptome data. DEGs were significantly enriched in cellular REDOX reactions and oxidoreductase activity when the *el-5y* mutant was yellowing, and most of these DEGs were downregulated in the *el-5y* mutants. Simultaneously, DEGs related to the homeostasis, transport, and binding of iron were also significantly downregulated in the *el-5y* mutant. Iron homeostasis is important for cell development, and iron transport and binding are closely related not only to cellular antioxidants but also to chloroplast development. These results indicated that the REDOX system in the *el-5y* mutant is defective, leading to the accumulation of harmful substances in the cell, thus impeding normal cell development. During the vegetative growth stage, the *el-5y* mutant can resist the damage caused by harmful substances by increasing SOD activity. However, as plants enter the reproductive growth stage, their developmental pattern changes, leading to the accumulation of harmful substances within the cells due to their inability to be cleared in time, resulting in yellowing and senescence.

The direct reason for etiolation is a decrease in the chlorophyll content caused by abnormal chloroplast development, either directly or indirectly (Liu et al., 2020). Although the mutant gene may not necessarily be related to chloroplast component proteins, genetic mapping of different leaf color materials helps us to comprehensively understand the mechanism of plant color formation. In this study, through the analysis of BSA-seq and RNA-seq, we mapped the candidate locus to a region of approximately 1.16 Mb on chromosome 11 and hypothesized *Glyma11G028200* as the candidate gene. *Glyma11G028200* encodes an ACS with only two non-synonymous mutations (ACT to AGT, and ACC to ATC, leading to Thr to Ser, and Thr to Ile, respectively) present in the functional domains of the *el-5y* mutant within the candidate region. Chloroplasts are the primary organelles responsible for auxin synthesis and ROS production (Yu et al., 2020; Hui et al., 2012). In plants, the mutations or overexpression of *ACS* genes may enhance the activity of NADPH oxidases through ethylene signaling, promoting the production of ROS including superoxide anions and hydrogen peroxide (Qu et al., 2017). Conversely, ROS can effect expression ACS gene (Wang et al., 2023). Meanwhile, IAA can induce ROS production in cells and promote lateral root development (Sandalio et al., 2016; Liu et al., 2017). Additionally, oxidative stress can enhance the auxin signaling pathway, and high levels of ROS can induce auxin accumulation (Biswas et al., 2019; Zou et al., 2019). The exogenous application of IAA can significantly increase ROS levels, thereby enhancing the activity of ethylene synthase (Liu et al., 1985; Ke and Sun, 2004; Naoki et al., 1991; Hansen and Grossmann, 2000). Notably, when the *el-5y* mutant exhibited a yellowing phenotype, DEGs related to auxin transport were significantly upregulated in the *el-5y* mutant. Based on the above information, we speculated that the mutations will affect the activity of ACS encoded by *Glyma11G028200*, which promotes the production of ROS through feedback, leading to further oxidative damage to the cells. At the same time, the auxin signaling pathway in the *el-5y* mutant becomes more active. Excessive ROS accumulation within cells disrupts normal cellular development and is accompanied by abnormal chloroplasts, resulting in plant yellowing and premature aging.

## Data Availability

The original contributions presented in this study are publicly available. The RNA-seq and BSA-seq datasets have been deposited in the NCBI BioProject database under accession numbers PRJNA1455432 and PRJNA1238100, respectively.

## Supplementary material

SUPPLEMENTARY FIGURE S1 Leaves damaged by insect herbivory **(A)** Wild-type VS-5, scale bars is 2cm; **(B)** the *el-5y* mutant, scale bars is 2cm.

SUPPLEMENTARY FIGURE S2 Correlation coefficients for samples. **A** and **B** are heat maps color represents the correlation coefficient; the numbers in the squares represent correlations between two samples, the closer the color of a square is to red, and the higher the correlation, the closer the color of a square is to blue, the lower the correlation. **(A)** Wild type (VS-5); **(B)** Mutant (*el-5y*); **(C)** Correlation analysis and hierarchical clustering of samples in leaves at the second node based on the reanalyzed dataset; **(D)** Principal component analysis (PCA) of samples in leaves at the second node based on the reanalyzed dataset.

SUPPLEMENTARY FIGURE S3 GO-Biological Process analysis of DEGs in different developmental stages between Wild-type and *el-5y* mutant. **(A)** DEGs from the leaves at the second node; **(B)** DEGs from the leaves at the fourth node; **(C)** DEGs from the leaves at the sixth node; **(D)** DEGs from fresh seeds.

SUPPLEMENTARY FIGURE S4 GO and KEGG analyses of DEGs in leaves at the second node based on the reanalyzed dataset. **(A)** Histogram presentation of Gene Ontology (GO) classifications of DEGs in leaves at the second node based on the reanalyzed dataset: y-axis indicates the number of DEGs in each subcategory. x-axis indicates the GO subcategories; **(B)** Histogram presentation of Kyoto Encyclopedia of Genes and Genomes (KEGG) classifications of DEGs in leaves at the second node based on the reanalyzed dataset: y-axis indicates the KEGG subcategories; the numbers in parentheses indicates the number of DEGs in each subcategory; x-axis and the numbers outside parentheses indicates percentage of DEGs in the subcategories; **(C)** GO-Biological Process analysis of of DEGs in leaves at the second node based on the reanalyzed dataset.

SUPPLEMENTARY FIGURE S5 GO analysis of 75 DEGs that exhibited a consistent expression pattern throughout all three leaf developmental phases.

SUPPLEMENTARY FIGURE S6 qRT-PCR Analysis of selected genes. The blue line represents value of FPKM (Fregments Per Kilobase per Million) and the red line represents the expression levels analyzed by qRT-PCR. The left column represents gene expression level in the wild type, the right column represents gene expression level in the mutant ts. Pearson correlation coefficients R range between 0.82 and 0.99. **(A, B)**: *Glyma.11G029400*; **(C, D)**: *Glyma.11G027800*, **(E, F)**: *Glyma.11G028200*, **(G, H)**: *Glyma.13G161900*, **(I, J)**: *Glyma.11G028600*.

SUPPLEMENTARY FIGURE S7 Genome-wide distribution of ΔSNP-index and Euclidean Distance (ED) values for BSA-seq analysis. **(A)** ΔSNP-index plot, **(B)** ED plot.

SUPPLEMENTARY FIGURE S8 Protein structure diagram of four mutant genes in candidate region. Black square: major domain, Orange square: low complexity, Blue square: transmembrane region, m: mutant site in protein, number: border of motif or domain in protein.
