# Supplementary figures and images for: Identification of a locus associated with chlorosis and antioxidant capacity using RNA-seq and BSA-seq in soybean [Glycine max (L.) Merr]
Source: Front Plant Sci. 2026 Jun 29;16:1598930. doi: 10.3389/fpls.2025.1598930 (PMC13358223; doi:10.3389/fpls.2025.1598930)

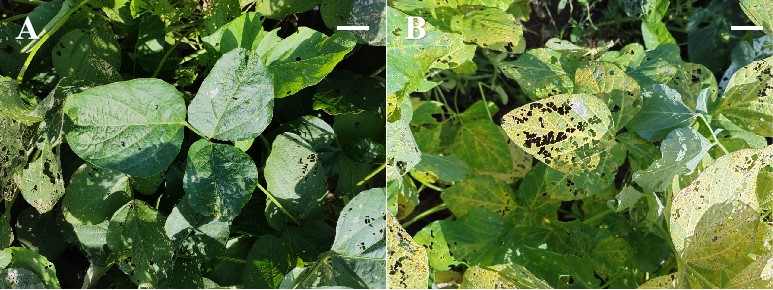

Supplement: SUPPLEMENTARY FIGURE S1 — Leaves damaged by insect herbivory (A) Wild-type VS-5, scale bars is 2cm; (B) the el-5y mutant, scale bars is 2cm. [file Image1.jpeg]

A

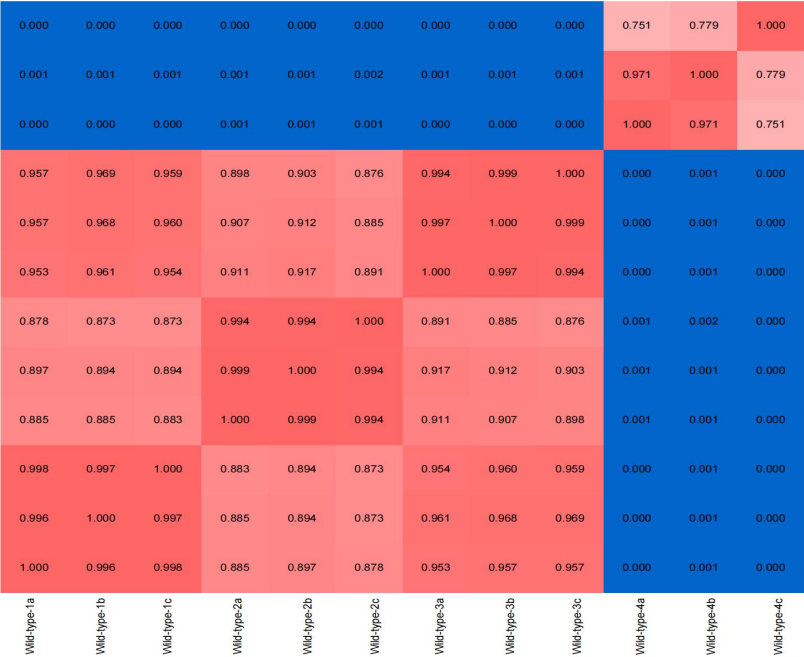

B

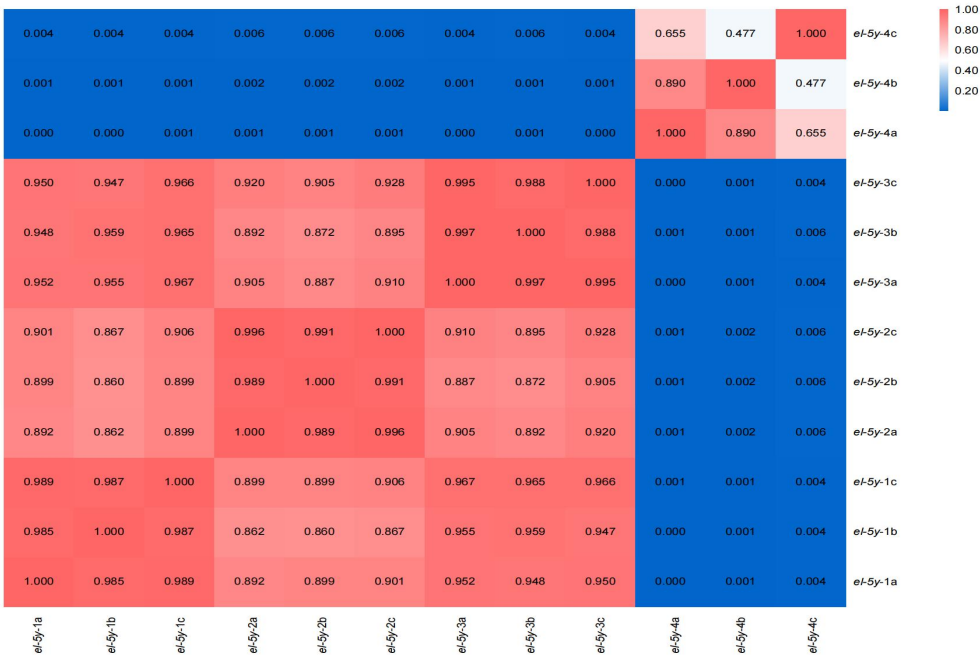

C

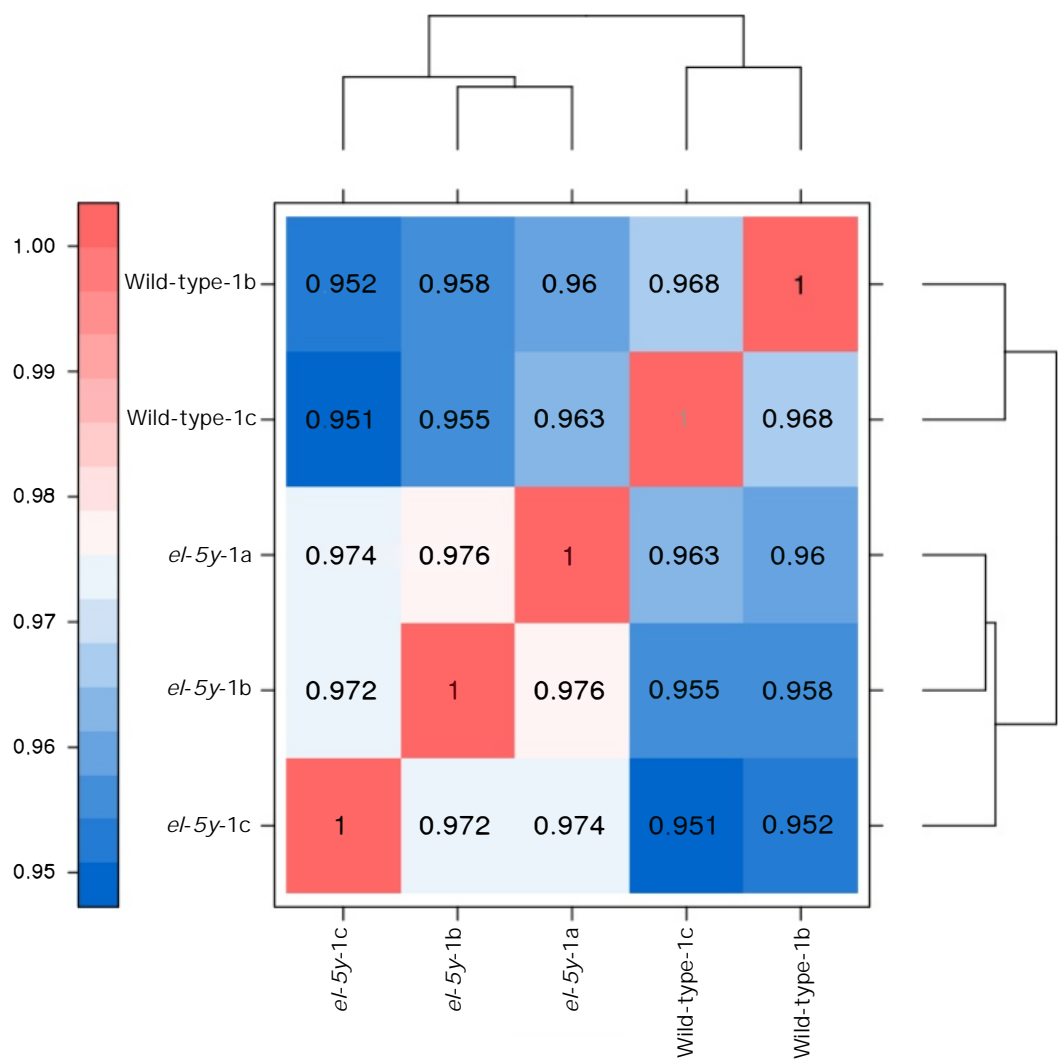

D

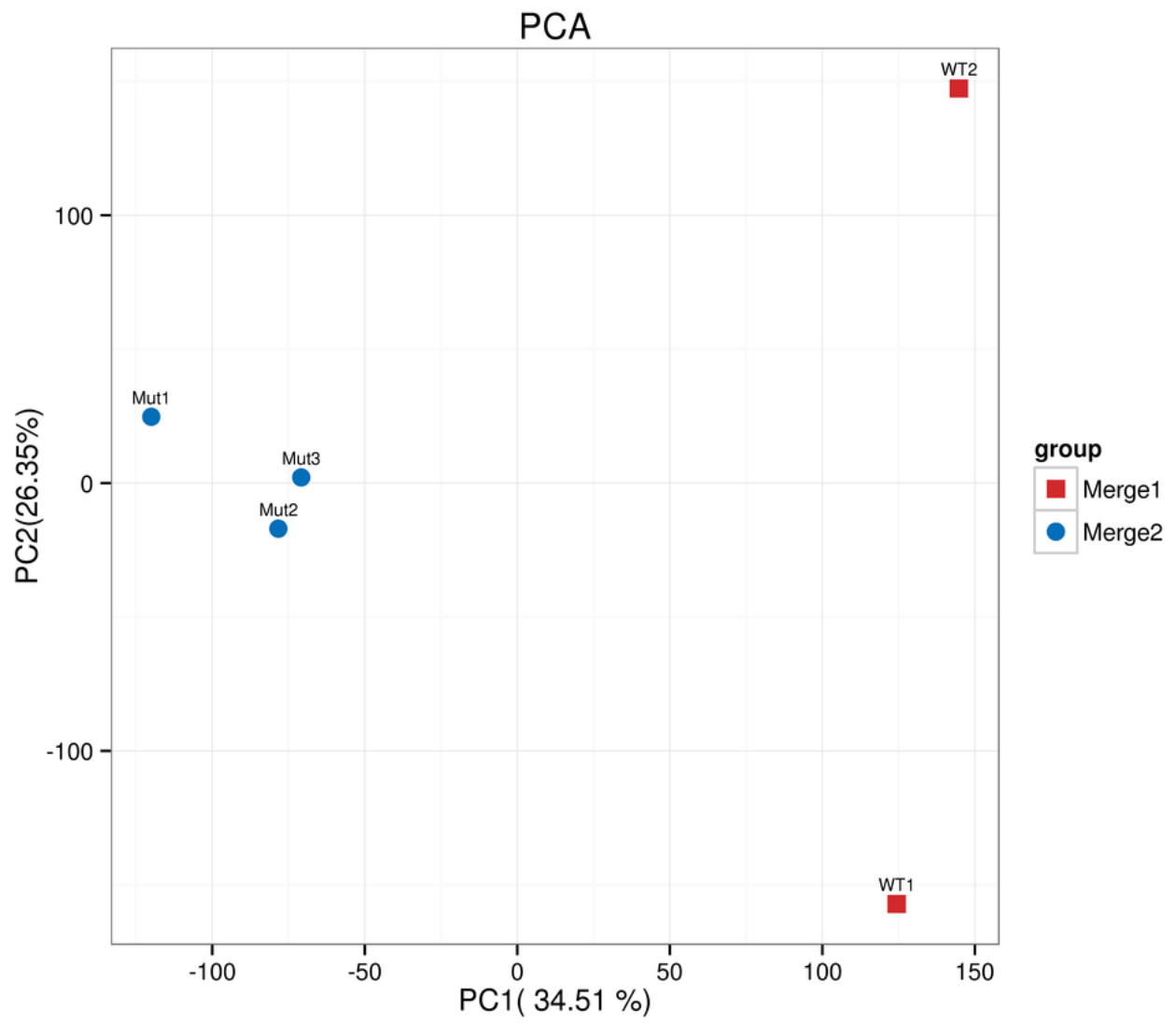

Supplement: SUPPLEMENTARY FIGURE S2 — Correlation coefficients for samples. A and B are heat maps color represents the correlation coefficient; the numbers in the squares represent correlations between two samples, the closer the color of a square is to red, and the higher the correlation, the closer the color of a square is to blue, the lower the correlation. (A) Wild type (VS-5); (B) Mutant (el-5y); (C) Correlation analysis and hierarchical clustering of samples in leaves at the second node based on the reanalyzed dataset; (D) Principal component analysis (PCA) of samples in leaves at the second node based on the reanalyzed dataset. [file Image2.pdf]

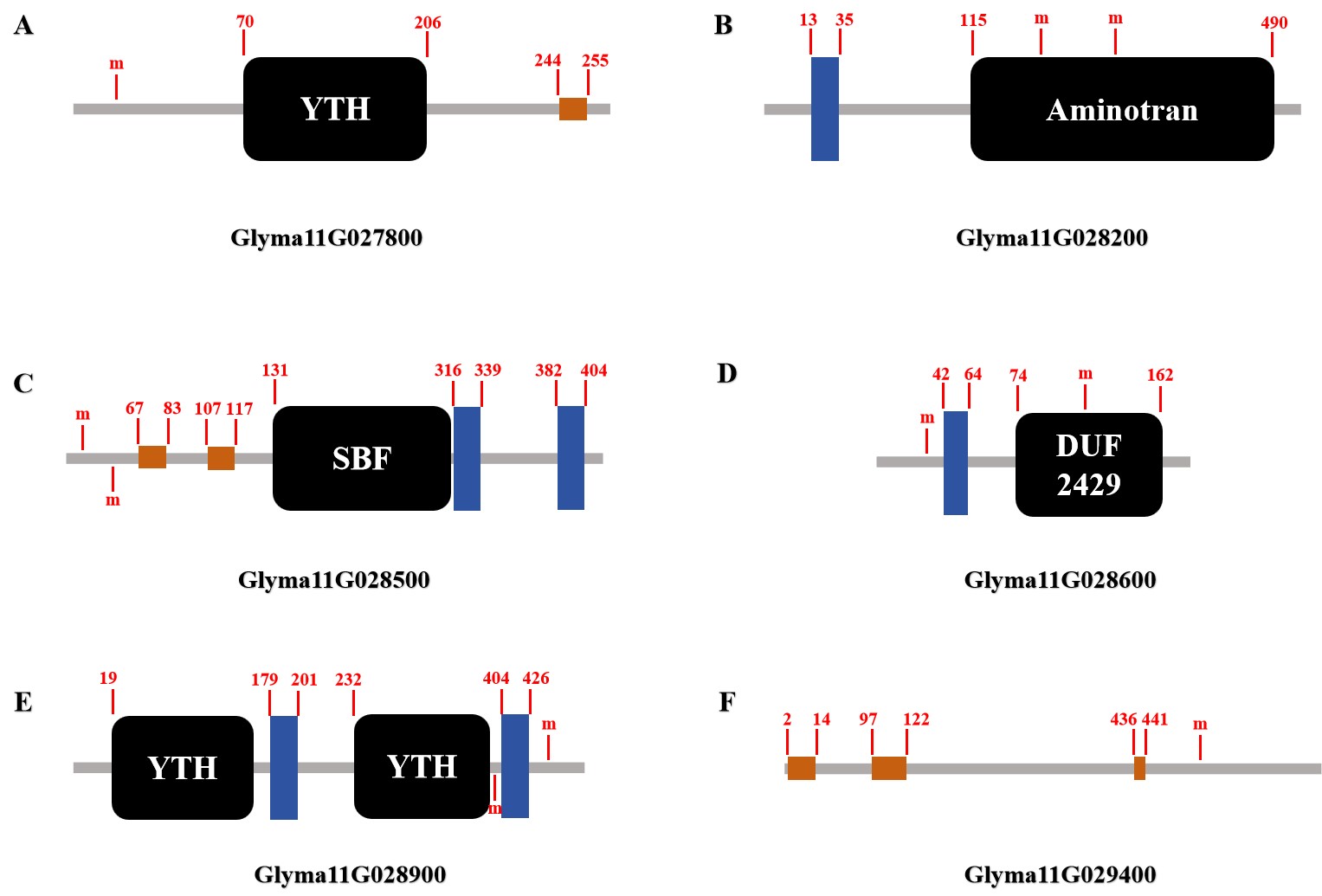

Supplement: SUPPLEMENTARY FIGURE S3 — GO-Biological Process analysis of DEGs in different developmental stages between Wild-type and el-5y mutant. (A) DEGs from the leaves at the second node; (B) DEGs from the leaves at the fourth node; (C) DEGs from the leaves at the sixth node; (D) DEGs from fresh seeds. [file Image3.jpeg]

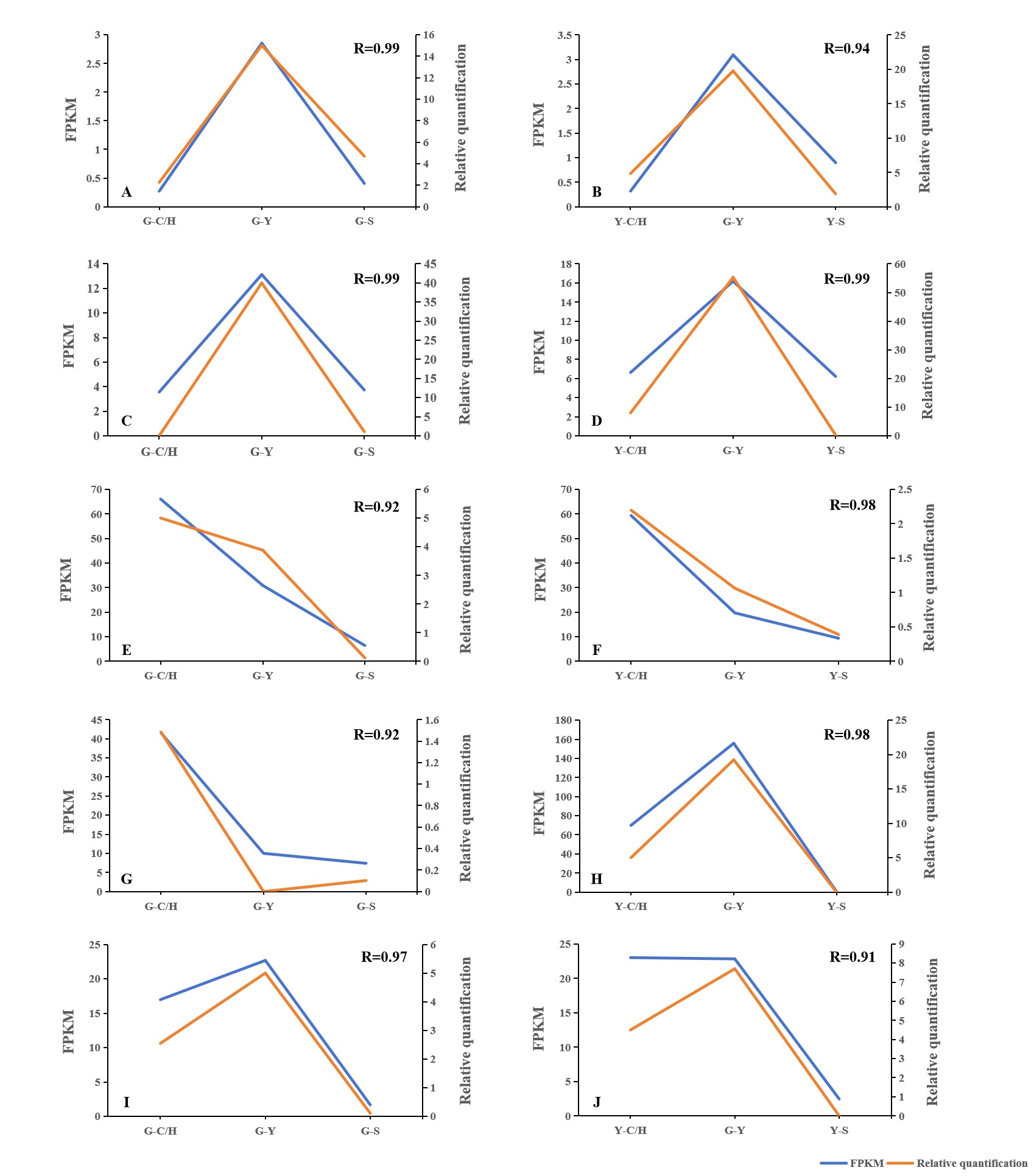

Supplement: SUPPLEMENTARY FIGURE S6 — qRT-PCR Analysis of selected genes. The blue line represents value of FPKM (Fregments Per Kilobase per Million) and the red line represents the expression levels analyzed by qRT-PCR. The left column represents gene expression level in the wild type, the right column represents gene expression level in the mutant ts. Pearson correlation coefficients R range between 0.82 and 0.99. (A, B): Glyma.11G029400; (C, D): Glyma.11G027800, (E, F): Glyma.11G028200, (G, H): Glyma.13G161900, (I, J): Glyma.11G028600. [file Image6.jpeg]

**A**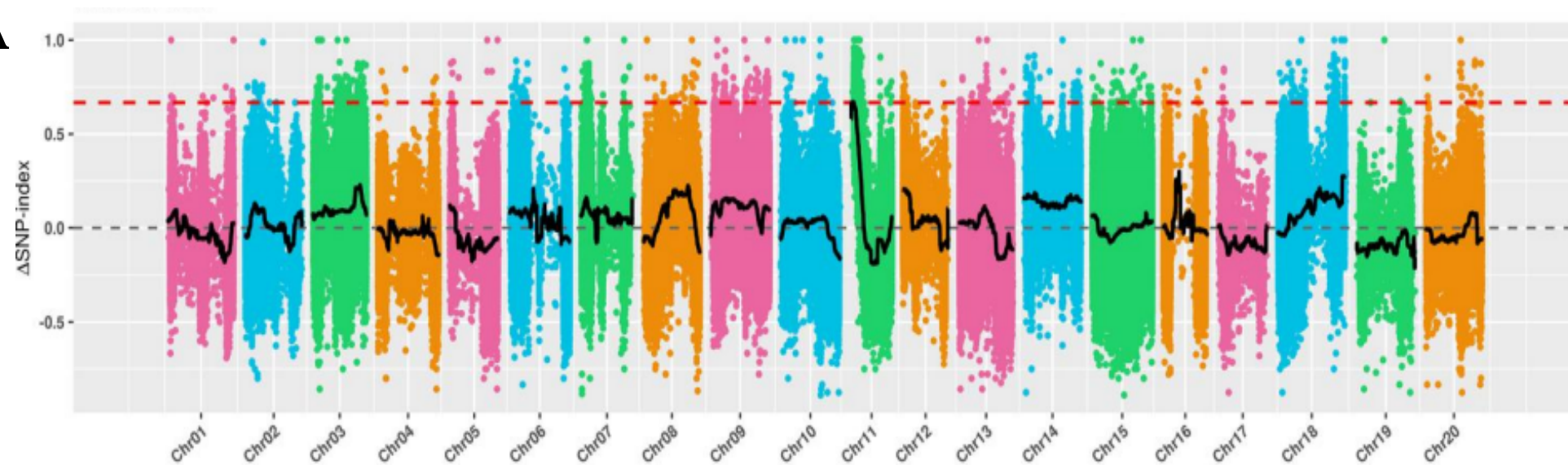**B**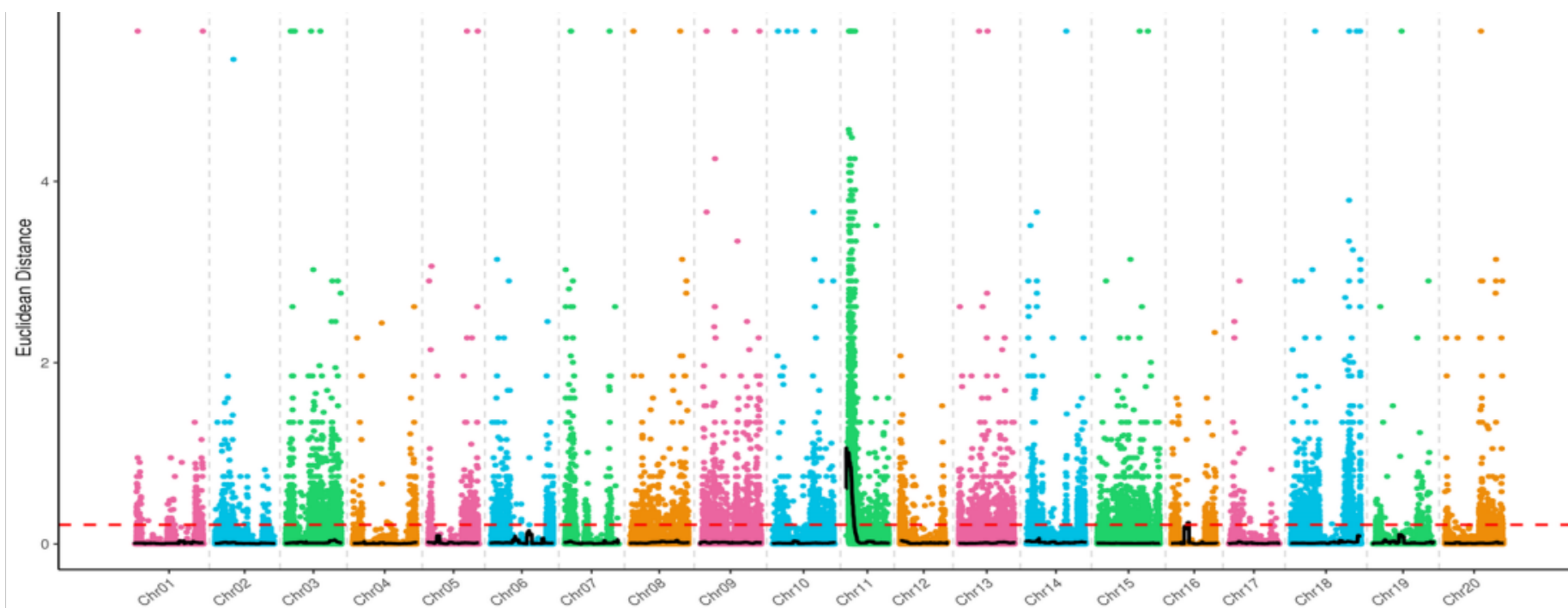

Supplement: SUPPLEMENTARY FIGURE S7 — Genome-wide distribution of ΔSNP-index and Euclidean Distance (ED) values for BSA-seq analysis. (A) ΔSNP-index plot, (B) ED plot. [file Image7.pdf]
