# Supplementary material for: Identification of a locus associated with chlorosis and antioxidant capacity using RNA-seq and BSA-seq in soybean [Glycine max (L.) Merr]
Source: Front Plant Sci. 2026 Jun 29;16:1598930. doi: 10.3389/fpls.2025.1598930 (PMC13358223; doi:10.3389/fpls.2025.1598930)
Supplement: SUPPLEMENTARY FIGURE S4 — GO and KEGG analyses of DEGs in leaves at the second node based on the reanalyzed dataset. (A) Histogram presentation of Gene Ontology (GO) classifications of DEGs in leaves at the second node based on the reanalyzed dataset: y-axis indicates the number of DEGs in each subcategory. x-axis indicates the GO subcategories; (B) Histogram presentation of Kyoto Encyclopedia of Genes and Genomes (KEGG) classifications of DEGs in leaves at the second node based on the reanalyzed dataset: y-axis indicates the KEGG subcategories; the numbers in parentheses indicates the number of DEGs in each subcategory; x-axis and the numbers outside parentheses indicates percentage of DEGs in the subcategories; (C) GO-Biological Process analysis of of DEGs in leaves at the second node based on the reanalyzed dataset. [file Image4.pdf]

A

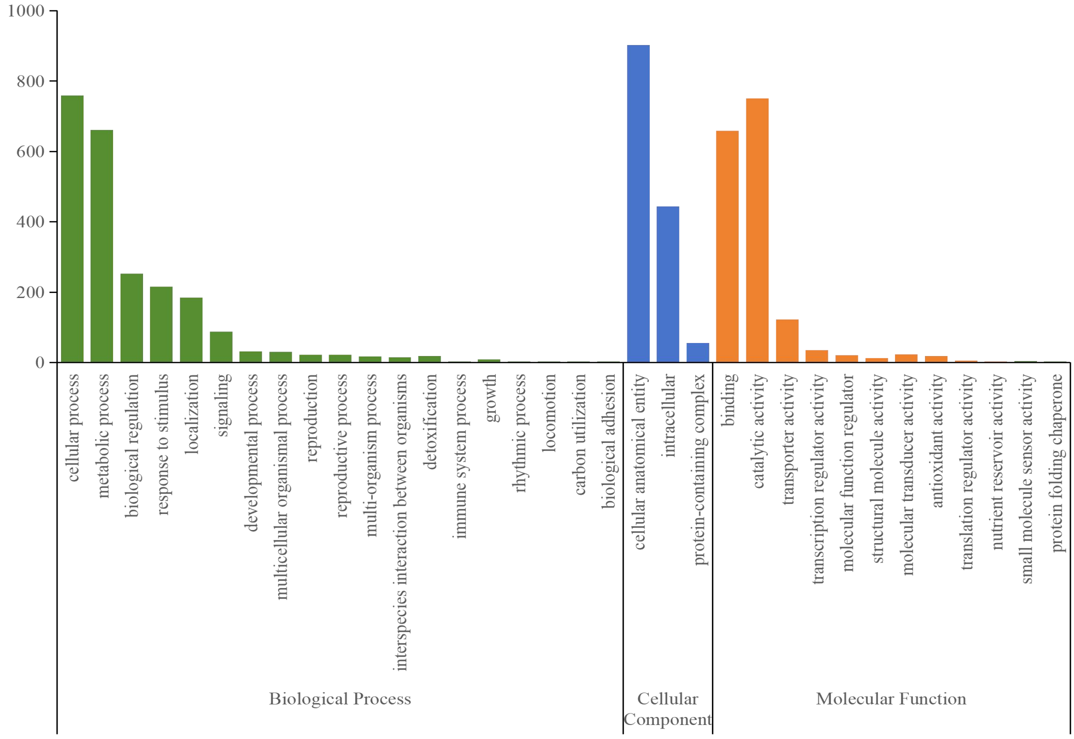

Histogram presentation of Gene Ontology (GO) classifications of DEGs in leaves at the second node based on the reanalyzed dataset

y-axis indicates the number of DEGs in each subcategory. x-axis indicates the GO subcategories.

B

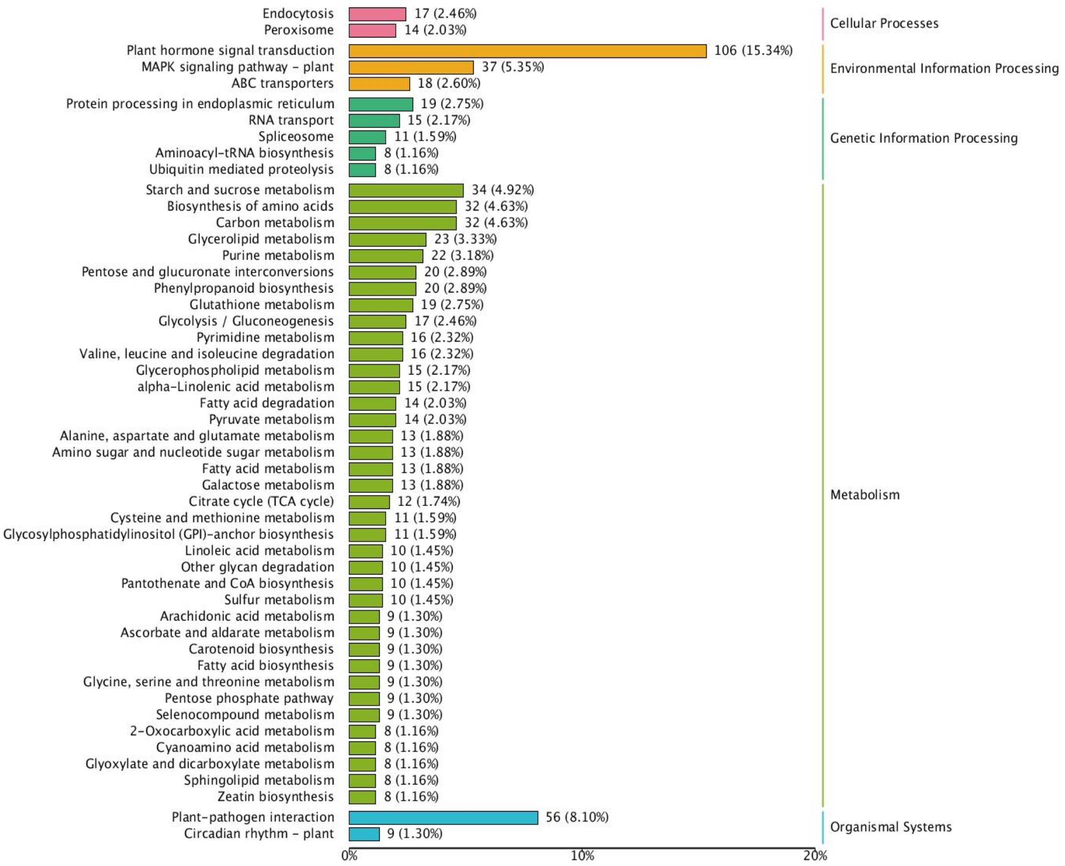

Histogram presentation of Kyoto Encyclopedia of Genes and Genomes (KEGG) classifications of DEGs in leaves at the second node based on the reanalyzed dataset

y-axis indicates the KEGG subcategories; the numbers in parentheses indicates the number of DEGs in each subcategory; x-axis and the numbers outside parentheses indicates percentage of DEGs in the subcategories.

C

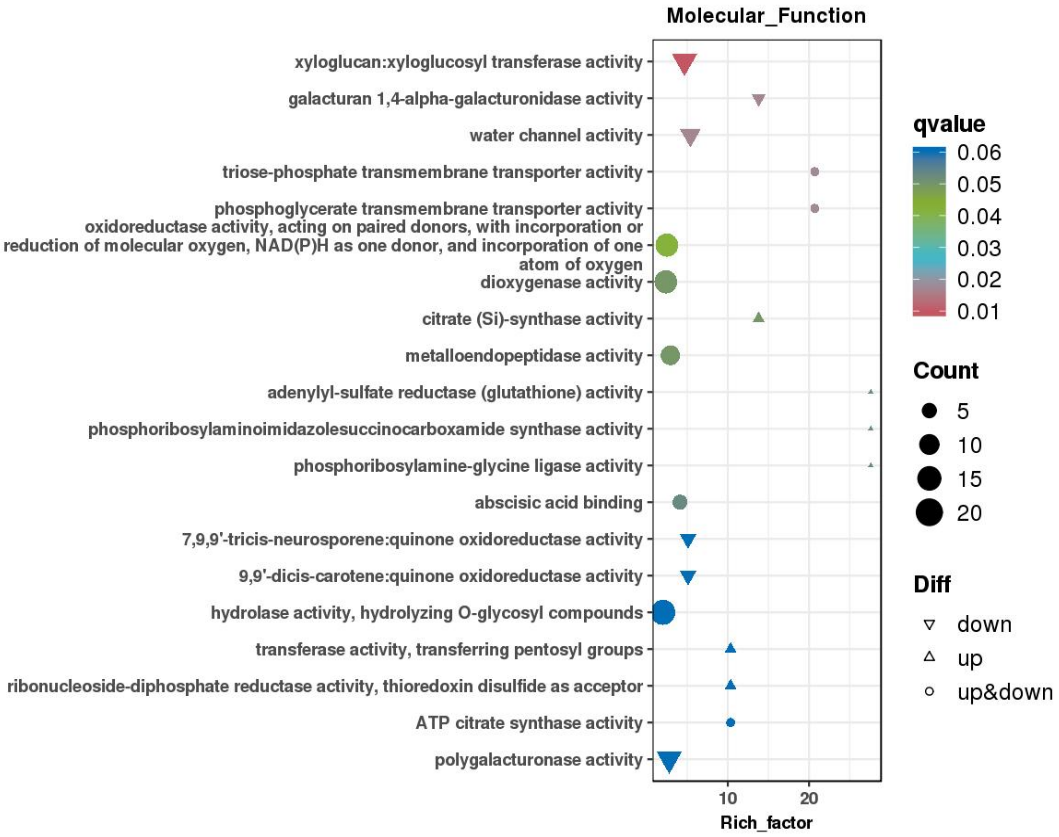

GO-Biological Process analysis of of DEGs in leaves at the second node based on the reanalyzed dataset
