# Supplementary material for: Identification of a locus associated with chlorosis and antioxidant capacity using RNA-seq and BSA-seq in soybean [Glycine max (L.) Merr]
Source: Front Plant Sci. 2026 Jun 29;16:1598930. doi: 10.3389/fpls.2025.1598930 (PMC13358223; doi:10.3389/fpls.2025.1598930)
Supplement: SUPPLEMENTARY FIGURE S5 — GO analysis of 75 DEGs that exhibited a consistent expression pattern throughout all three leaf developmental phases. [file Image5.pdf]

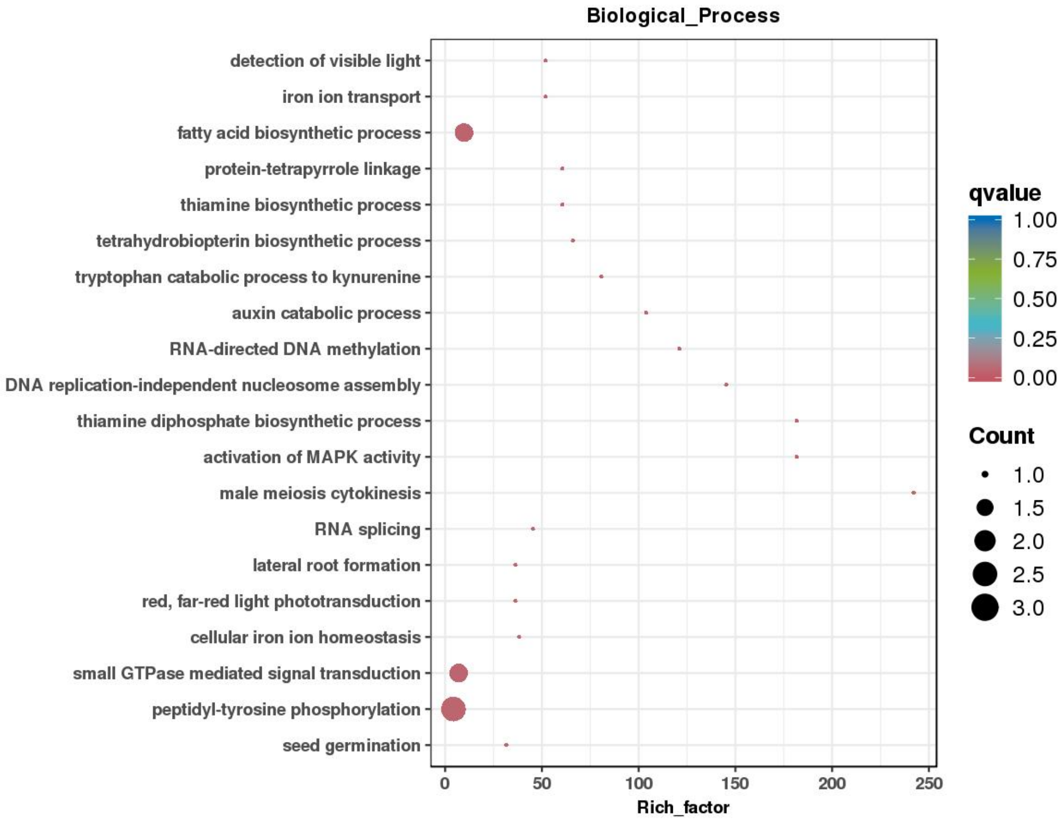

GO-Biological Process analysis of 75 DEGs that exhibited a consistent expression pattern throughout all three leaf developmental phases

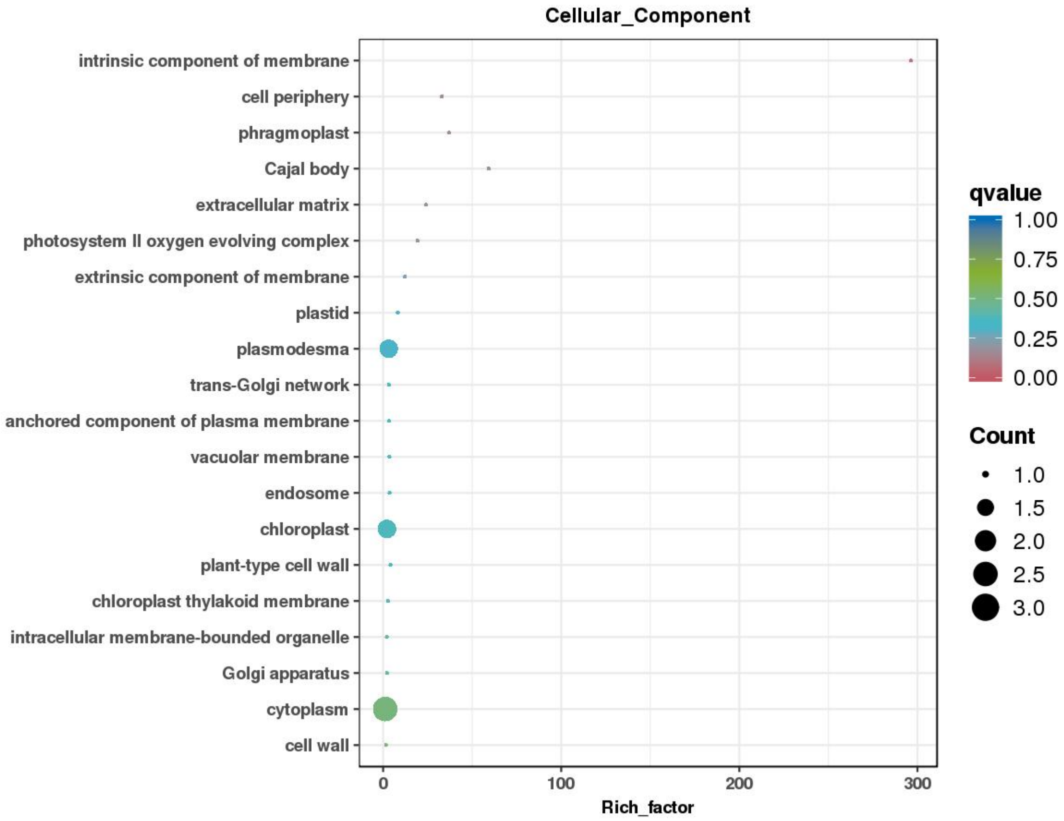

GO-Cellular component analysis of 75 DEGs that exhibited a consistent expression pattern throughout all three leaf developmental phases

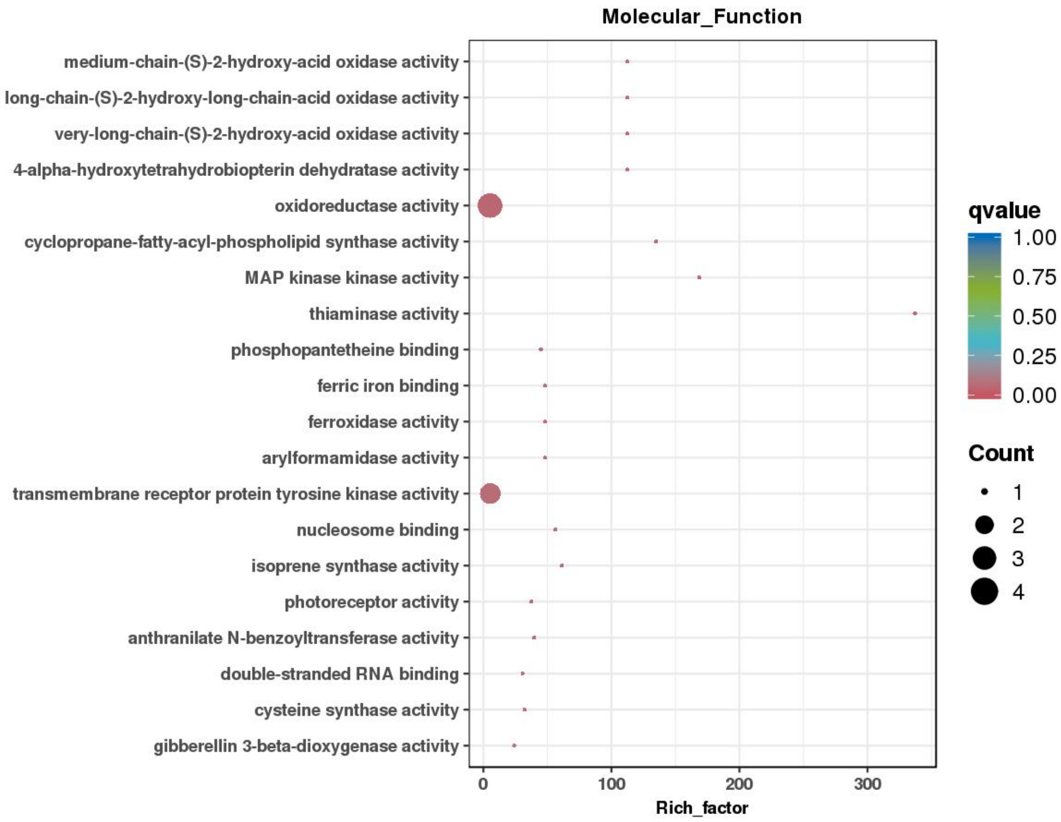

GO-Molecular Function analysis of 75 DEGs that exhibited a consistent expression pattern throughout all three leaf developmental phases
